# Mitochondria and aging: A role for the mitochondrial transition pore?

**DOI:** 10.1111/acel.12793

**Published:** 2018-06-11

**Authors:** Mathieu Panel, Bijan Ghaleh, Didier Morin

**Affiliations:** ^1^ INSERM U955, équipe 3 Créteil France; ^2^ Université Paris‐Est, UMR_S955, DHU A‐TVB, UPEC Créteil France

**Keywords:** age‐associated diseases, aging, calcium, mitochondria, mPTP, oxidative stress

## Abstract

The cellular mechanisms responsible for aging are poorly understood. Aging is considered as a degenerative process induced by the accumulation of cellular lesions leading progressively to organ dysfunction and death. The free radical theory of aging has long been considered the most relevant to explain the mechanisms of aging. As the mitochondrion is an important source of reactive oxygen species (ROS), this organelle is regarded as a key intracellular player in this process and a large amount of data supports the role of mitochondrial ROS production during aging. Thus, mitochondrial ROS, oxidative damage, aging, and aging‐dependent diseases are strongly connected. However, other features of mitochondrial physiology and dysfunction have been recently implicated in the development of the aging process. Here, we examine the potential role of the mitochondrial permeability transition pore (mPTP) in normal aging and in aging‐associated diseases.

## 1 INTRODUCTION

Aging is a physiological process occurring over life that induces a general decline of physical and mental capacities. Despite numerous studies, the mechanisms of aging remain to be established. Aging is associated with dysfunction of organs and alteration of their performance, such as hearing failing or muscle weakness, which can lead to a loss of independence but also to the development of diseases. Therefore, a better knowledge of the mechanism of aging would contribute to improve the quality of life of the elderly. Several theories have been proposed to explain the mechanisms of aging (Allison et al., 2014; Park & Yeo, 2013). They include among others genetic predisposition, programmed senescence, DNA damage, endocrine dysfunction, or the free radical hypothesis. It is likely that the mechanisms described in these theories may participate to those of aging but none of them can directly explain the causes of aging. Another theory centered on mitochondrial dysfunction was proposed half a century ago (Harman, 1972). This theory is closely linked to the free radical hypothesis of aging but also involves genetic and bioenergetic alterations. Mitochondria are central organelles in the cell. They are present in all cells of humans and animals (except red blood cells). They generate cellular energy, produce reactive oxygen species (ROS) that regulate physiological processes (Angelova & Abramov, 2016), and are involved in the control of cell death (Galluzzi, Kepp, Trojel‐Hansen, & Kroemer, 2012). Therefore, it is not surprising that mitochondria could be involved in the normal mammalian aging process. One of the unique characteristics of mitochondria is that they possess their own genetic material in the form of a close circular DNA molecule. According to this latter theory, aging of cells would be due to the constant delivery of ROS inside mitochondria throughout life, damaging mitochondrial DNA which is vulnerable as it is not protected by protein histones or repairing enzymes such as nuclear DNA. The damaged mitochondrial DNA leads to deficiency of key electron transport enzymes and subsequent ROS generation, thus causing a vicious cycle of ROS resulting in a decrease in energy production (Fariss, Chan, Patel, Van Houten, & Orrenius, 2005).

Although a large amount of data support the role of mitochondrial ROS production in aging, other features of mitochondrial physiology and dysfunction, including the mitochondrial permeability transition, have been more recently implicated in the mechanisms of aging (Balaban, Nemoto, & Finkel, 2005; Bratic & Larsson, 2013; Gonzalez‐Freire et al., 2015; Payne & Chinnery, 2015).

The mitochondrial permeability transition has been characterized by the pioneering work of Hunter and Haworth and corresponds to the sudden increase in the permeability of the inner mitochondrial membrane to molecules of molecular mass up to 1,500 Da (Haworth & Hunter, 1979; Hunter & Haworth, 1979a, 1979b). The opening is due to a nonspecific pore called the mitochondrial permeability transition pore (mPTP) occurring when mitochondria become overloaded with calcium. The sensitivity of the mPTP to calcium is enhanced under oxidative stress conditions, adenine nucleotide depletion, high phosphate concentrations, or membrane depolarization (Halestrap & Richardson, 2015). mPTP opening induces swelling of the organelle matrix, collapse of membrane potential, and uncoupling of oxidative phosphorylation (Crompton, 1999). This phenomenon plays a critical role in different types of cell death. Although the conditions leading to permeability transition are well known, the exact composition of the pore remains unknown. Many proteins were thought to form the core of the pore across the mitochondrial membrane but they have been successively ruled out by genetic modulation. This is the case for the voltage‐dependent anion channel (VDAC) and the translocator protein (TSPO) in the outer membrane (Baines, Kaiser, Sheiko, Craigen, & Molkentin, 2007; Kokoszka et al., 2004). Recent data propose a role for ATP synthase as the major component of a multiproteic complex (Bernardi, Rasola, Forte, & Lippe, 2015). Currently, a common agreement considers that cyclophilin D (CypD), a soluble protein located within the mitochondrial matrix, is the main partner of the mPTP (Gutiérrez‐Aguilar & Baines, 2015) and that mPTP formation is greatly sensitized by CypD which lowers the calcium threshold required to trigger mPTP opening. The crucial role of CypD has been shown by deletion of the gene in mice, allowing mitochondria to sustain high calcium concentrations and thus conferring major desensitization of mPTP (Baines et al., 2005). Two opening states of the pore have been distinguished, a permanent or long‐lasting state which is associated with cell death, and a transient opening state having a physiological role by providing a pathway to release ROS and calcium from mitochondria which is also regulated by CypD (Elrod et al., 2010; Hausenloy, Wynne, Duchen, & Yellon, 2004; Petronilli et al., 1999). The mPTP is now considered to be central in numerous conditions such as heart, brain, or liver ischemia–reperfusion (Friberg & Wieloch, 2002; Halestrap, 2010; Kim, He, Qian, & Lemasters, 2003; Morin, Hauet, Spedding, & Tillement, 2001; Rauen & de Groot, 2004), drug‐induced liver injury (Jaeschke, McGill, & Ramachandran, 2012), age‐related neurodegenerative diseases (Rao, Carlson, & Yan, 2014), and accumulating data imply the mPTP in organ dysfunction occurring during aging (Hepple, 2016; Rocha‐Rodrigues et al., 2013; Toman & Fiskum, 2011). Conversely, caloric restriction, which is a proven strategy to delay aging and age‐related disease (Balasubramanian, Howell, & Anderson, 2017), is associated with the inhibition of mPTP opening (Amigo, Menezes‐Filho, Luévano‐Martínez, Chausse, & Kowaltowski, 2017; Hofer et al., 2009; Kristal & Yu, 1998; Menezes‐Filho et al., 2017).

The aim of this review is to summarize the current data showing a relationship between mPTP opening and aging. We will analyze this relationship through the alterations of the cellular stimuli involved in the two processes, the modification of the proteins that are considered as components of the pore and finally, we will show that mPTP opening is involved in the occurrence of different pathologies during aging.

## 2 EXPERIMENTAL EVIDENCE SUPPORTING THE INVOLVEMENT OF THE mPTP DURING AGING

Mitochondrial dysfunction is considered as a main feature of aging (Bratic & Larsson, 2013; López‐Otín, Blasco, Partridge, Serrano, & Kroemer, 2013). When a cell ages, the efficiency of oxidative phosphorylation decreases, reducing ATP production. This impairs mitochondrial function and results in an aging phenotype, more particularly in organs requiring a high energy supply such as the heart, muscles, brain, or liver. The regulation of mPTP opening is also altered by aging as demonstrated in mitochondria isolated from various aged or senescent tissues. This may be related to the similarity of the stimuli involved in mPTP opening and cellular aging.

An increased sensitivity to calcium overload was observed in mitochondria isolated from senescent rat heart (Fernandez‐Sanz et al., 2015; Jahangir, Ozcan, Holmuhamedov, & Terzic, 2001; Ljubicic, Menzies, & Hood, 2010; Petrosillo, Moro, Paradies, Ruggiero, & Paradies, 2010). This effect was confirmed in permeabilized cardiomyocytes (Picard, Wright, Ritchie, Thomas, & Hepple, 2012) but may be restricted to interfibrillar mitochondria (Fernandez‐Sanz et al., 2015; Hofer et al., 2009). An enhanced susceptibility to mPTP opening was also found in brain (Krestinina et al., 2015; Marques‐Aleixo et al., 2012; Mather & Rottenberg, 2000) and appeared to depend on the brain area tested (Brown, Geddes, & Sullivan, 2004; LaFrance, Brustovetsky, Sherburne, Delong, & Dubinsky, 2005), in the liver (Goodell & Cortopassi, 1998; Mather & Rottenberg, 2000), and in lymphocytes (Rottenberg & Wu, 1997). More recently, Picard, Ritchie, Thomas, Wright, and Hepple (2011) described an impaired mPTP function with aging in fast muscles of the rat that was also observed in aged human muscles (Gouspillou et al., 2014), showing that this phenomenon is not restricted to animal models of aging. Sensitization of mPTP opening was also involved in the bone loss occurring in aging mice (Shum et al., 2016). However, it should be kept in mind that most of these data were obtained in isolated mitochondria, which may amplify mitochondrial functional impairment (Picard et al., 2010).

Other studies also showed that mPTP regulation is dysfunctional in the aged myocardium. Indeed, pharmacological mPTP inhibitors failed to produce significant effects in either normal or stressed conditions. For instance, cyclosporin A (CsA) was unable to inhibit carboxyatractyloside‐induced permeability transition in aged mitochondria (García, Zazueta, Martínez‐Abundis, Pavón, & Chávez, 2009) and to prolong the time necessary to induce mPTP opening in isolated mitochondria (Duicu et al., 2013) and in cardiomyocytes isolated from old rats (Liu, Zhu, Brink, Glass, & Rebecchi, 2011). Similarly, the ability of sevoflurane and isoflurane conditioning (Li et al., 2013; Zhu et al., 2010) and of the GSK‐3β inhibitor SB‐216763 (Zhu, Rebecchi, Glass, Brink, & Liu, 2011) to protect against myocardial ischemia–reperfusion injury, which is mediated by inhibition of mPTP opening in young rats, is abrogated in senescent animals. Taken together, these data support the hypothesis of mPTP activation during aging in rodents. This is consistent with the increased apoptotic susceptibility observed in different organs (Chabi et al., 2008; Kwak, Song, & Lawler, 2006), although mechanisms other than mPTP activation for the induction of apoptosis have been proposed (Chabi et al., 2008).

## 3 REGULATING FACTORS OF mPTP AND AGING

### 3.1 Calcium homeostasis, mPTP, and aging

Elevated matrix calcium was the first factor described to activate mPTP opening (Haworth & Hunter, 1979). Although calcium overload is still considered as essential, other factors, such as oxidative stress, adenine nucleotide depletion, or high phosphate concentrations, are also involved in the formation and/or in the regulation of the pore. These factors enhance the sensitivity of the mPTP to calcium that possesses binding sites in the mitochondrial inner membrane facing the matrix (Halestrap & Richardson, 2015). Therefore, the level of cellular calcium can influence mPTP occurrence. Aging alters cytosolic calcium handling. This has been clearly demonstrated in the heart where aging impairs the myocardial calcium transport system, calcium storage capacities, and contractile function (Besse et al., 1994; Feridooni, Dibb, & Howlett, 2015; Frolkis et al., 1988; Kaplan et al., 2007). This was recently confirmed in myocytes isolated from human right atria (Herraiz‐Martínez et al., 2015) and suggests a progressive decline in right atrial contractile function with age.

The enhancement of basal calcium levels can promote the activation of calcium‐dependent enzymes such as phospholipases, proteases, and nucleases and can alter oxidative phosphorylation (Jahangir et al., 2001). This may predispose mitochondria to calcium overload and therefore to mPTP opening. This phenomenon is reinforced under stress such as ischemia–reperfusion (Jahangir, Sagar, & Terzic, 2007). Aging also impairs calcium communication between sarcoplasmic reticulum and mitochondria which are tightly interconnected in cardiac cells (Szalai, Csordás, Hantash, Thomas, & Hajnóczky, 2000). This defective communication alters calcium transfer and contributes to a deficiency in energy production and to an increase in oxidative stress in the aged heart (Fernandez‐Sanz et al., 2014). A similar mechanism was observed in heart failure and may be involved in the initiation and progression of the disease (Kohlhaas & Maack, 2013).

Disturbances in calcium regulation and mitochondrial homeostasis may also contribute to the decline of muscle performance in aging (for review, see Del Campo, Jaimovich, & Tevy, 2016). Indeed, Pietrangelo et al. (2015) described an age‐related structural uncoupling between calcium release units and mitochondria which could impair the control of calcium levels in muscle and consequently the efficiency of ATP synthesis. The dysregulation of neuronal calcium homeostasis has also been identified as playing an important role in the process of normal aging in brain. This “calcium hypothesis of neuronal aging” has evolved over time, and the concomitant perturbations of cellular calcium, mitochondrial function, and oxidative stress are now considered to participate to the neuron degeneration occurring during aging (Toescu & Vreugdenhil, 2010). Whether mPTP opening is involved in this process remains an open question. However, recent data showed that aging reduced ATP synthesis and mitochondrial calcium buffering capacities and increased the sensitivity of mPTP formation in the putamen of aged monkeys. This was correlated with a reduction in locomotor activity compared with younger animals (Pandya et al., 2015).

**Figure 1:**
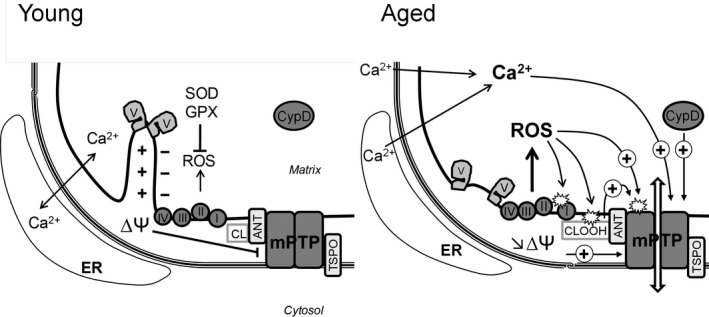
Reactive oxygen species (ROS), calcium, membrane potential, and mPTP opening during aging. In mitochondria from young animals, mPTP opening is prevented by the high membrane potential (∆Ψ), the regulation of the matrix calcium concentration, and ROS detoxification Aging is characterized by loss of cristae structure due to disassembly of ATP synthase dimers, increased calcium content, and ROS production as well as decline in membrane potential. Alteration in calcium handling results in elevated matrix calcium which is the primary trigger for mPTP opening. Mitochondrial respiratory chain is the main producer and target of ROS. ROS have multiple targets including respiratory chain complexes, leading to defective complexes producing more ROS and lowering membrane potential in a vicious circle. ROS production also promotes cardiolipin (CL) peroxidation (CLOOH) which sensitizes mPTP to calcium overload. Translocator protein (TSPO) and adenine nucleotide translocase (ANT) might also play a role in mPTP opening during aging. ER, endoplasmic reticulum; SOD, superoxide dismutase; GPX, glutathione peroxidase; ┴, inhibition; (+)→, stimulation

Taken together, these data indicate that the disturbance of calcium cellular homeostasis may contribute to the aging process, more particularly in the excitable cells. Increasing evidence suggests that this alteration can affect mitochondrial energy production and promote oxidative stress (Figure 1). However, the available information does not allow to draw definite conclusions on a possible role of calcium dysregulation in the occurrence of mPTP opening in healthy aging, although calcium is a major actor in the induction of mPTP opening.

### 3.2 ROS generation, mPTP, and aging

It is well known that mitochondria are producers of ROS. The electron leakage in the electron transport chain during respiration is generally considered as the main source of mitochondrial ROS but other mitochondrial enzymatic systems, such as monoamine oxidase and cytochrome b5 reductase in the outer membranes, cytochromes P450 enzymes in the inner membranes, or several matrix enzymes such as aconitase, can also produce ROS (Andreyev, Kushnareva, Murphy, & Starkov, 2015; Andreyev, Kushnareva, & Starkov, 2005). Although mitochondria are not always considered as the main producer of ROS in the cell (NADPH or xantine oxidases being able to produce high levels of ROS), the respiratory chain produces ROS continuously. Reactive oxygen species were initially considered to be toxic molecules but a growing body of evidence suggests that oxidative stress, which is the result of a balance between the formation of ROS and their scavenging by antioxidant defenses, is regulated and participates to the maintenance of redox homeostasis and various cellular signaling pathways. In normal cells, the cellular and mitochondrial levels of ROS are safe and participate to the vital activity of the cell (Angelova & Abramov, 2016; Bae, Oh, Rhee, & Yoo, 2011; Dröge, 2002; Nickel, Kohlhaas, & Maack, 2014). However, under acute and chronic cellular stress conditions (e.g., acute ischemia and neurodegenerative diseases, respectively), the production of ROS is no longer regulated and becomes detrimental for the cell. Evidence also suggests that aging involves a change in ROS regulatory processes encompassing a decline in mitochondrial function and an increase in ROS generation (Brand, 2014; Bratic & Larsson, 2013; Skulachev & Skulachev, 2014). For instance, monoamine oxidase activity in 24‐month‐old rat cardiac mitochondria was much stronger than that in 1‐month‐old rats, showing that monoamine oxidase may be an important source of ROS in the aging heart (Di Lisa, Kaludercic, Carpi, Menabo, & Giorgio, 2009; Maurel et al., 2003). In this context, an interesting relationship was found between the rate of ROS production during mitochondrial reverse electron transport in vitro and lifespan in vertebrate homeotherms (Lambert et al., 2007). Several reviews have described the mechanisms of ROS production in mitochondria and discussed their potential contribution in aging (Balaban et al., 2005; Brand, 2010). Here, we will only give a brief summary and we will focus on the possible link between ROS production and mPTP opening during aging.

Mitochondrial ROS are primarily the result of the inefficient transfer of electrons through the electron transfer chain, and this effect was reported to increase with age. This was assigned to the decline in the electron transfer chain capacity, the dysfunction of respiratory complexes, the decrease in ROS scavenging enzymes, and the induction of mutations of mitochondrial DNA, which is susceptible to oxidative damage because it lacks protection from ROS and because of its proximity to them (Balaban et al., 2005; Genova & Lenaz, 2015; Hoppel, Lesnefsky, Chen, & Tandler, 2017; Kwon, Choi, Cho, & Lee, 2015). It was suggested that the accumulation of these mutations in turn deteriorates electron transfer chain function and further increases ROS production, leading to a deleterious vicious cycle. These data are the basis of the mitochondrial free radical theory of aging. Numerous data argue in favor of the central role of ROS in aging, and a progressive mitochondrial dysfunction with increased levels of oxidized lipids and proteins is always considered a hallmark of aging (Skulachev & Skulachev, 2014). The beneficial effects of mitochondria‐targeted drugs such as plastoquinone derivatives (Anisimov et al., 2008, 2011) or MitoTEMPO (Miura et al., 2017; Owada et al., 2017) and of endogenous indoleamine melatonine (Escames et al., 2010; Paradies, Paradies, Ruggiero, & Petrosillo, 2017) reinforce this theory. Similarly, mice deleted for the gene of the p66shc adaptor protein have reduced ROS generation and delayed aging (Migliaccio et al., 1999; Napoli et al., 2003). However, new recent data challenged this hypothesis as they show that ROS can be beneficial and extend lifespan at least in lower organisms such as flies and worms (Sanz, 2016; Sena & Chandel, 2012). Accumulating evidence suggests that other aspects of mitochondrial physiology must be considered to explain the contribution of mitochondria to aging (Gonzalez‐Freire et al., 2015; Payne & Chinnery, 2015).

Reactive oxygen species decrease the calcium concentration needed for mPTP opening and thus sensitize mPTP opening (Figure 1). The increased formation of ROS and the oxidation of mitochondrial membrane lipids and proteins associated with mPTP are thus likely to promote mPTP opening during aging. This is what was observed with cardiolipin, a phospholipid that is specific of mitochondria and plays a major role in the molecular organization and the function of the inner mitochondrial membrane, interacting with many proteins (Klingenberg, 2009; Schlame & Greenberg, 2017). As cardiolipin is located close to the sources of ROS production and contains high level of unsaturated fatty acids, it is susceptible to lipid peroxidation. Oxidized cardiolipin was shown to sensitize heart mitochondria to mPTP opening (Petrosillo, Casanova, Matera, Ruggiero, & Paradies, 2006). The level of cardiolipin diminishes with age and that of oxidized cardiolipin increases. This has been suggested to be one of the mechanisms responsible for the alteration of the biochemical function of mitochondrial membranes (Paradies, Paradies, Ruggiero, & Petrosillo, 2014). A relevant hypothesis is that the oxidation of cardiolipin might sensitize mPTP opening to calcium during aging. In accordance with this hypothesis, Petrosillo et al. (2010) demonstrated that the ability of mitochondria to retain calcium, a marker of mPTP sensitivity, is altered during aging. The mechanism may involve the adenine nucleotide translocase (ANT). Indeed, cardiolipin interacts with and plays a key role for the transport of adenine nucleotides by ANT (Hoffmann, Stöckl, Schlame, Beyer, & Klingenberg, 1994), which has long been considered as a structural component of the mPTP. Although deletion experiments have challenged this hypothesis (Kokoszka et al., 2004), additional data demonstrate a role of ANT in facilitating mPTP opening (Halestrap & Richardson, 2015). The oxidation of cardiolipine, which is tightly bound to ANT, could modify its conformation and facilitate mPTP opening.

Oxidation–reduction of critical protein residues could also influence mPTP opening (Chernyak & Bernardi, 1996). More particularly, the oxidation of thiol functions and cysteine residues, which is an important mechanism regulating protein structure, was reported on proteins described to be involved in the formation of the pore or in the regulation of its opening, such as ANT (Costantini et al., 2000; Halestrap, Woodfield, & Connern, 1997), CypD (Nguyen et al., 2011), ATP synthase (Wang, Murray, Chung, & Van Eyk, 2013), or complex I of the respiratory chain (Chouchani et al., 2013). As both glutathione and cysteine systems become oxidized during aging (Go & Jones, 2017), this can contribute to mPTP opening. For instance, ANT contains three redox‐sensitive cysteines that are particularly prone to oxidation during aging (Yan & Sohal, 1998).

### 3.3 Membrane potential, mPTP, and aging

Several studies have shown that the mitochondrial membrane potential is lower in aged cells (Sastre et al., 1996; Sugrue & Tatton, 2001). This may have consequences on mPTP opening as mPTP is a voltage‐dependent channel which tends to open upon depolarization (Figure 1). In vitro, depolarization induces mPTP opening when mitochondria have been suitably loaded with calcium (Scorrano, Petronilli, & Bernardi, 1997). The reason for this decrease in mitochondrial membrane potential during aging is unknown but the enhancement of ROS formation, which is likely to modify mitochondrial membrane components and to promote mitochondrial uncoupling, is probably involved. Indeed, oxidative stress was shown to alter the fluidity and the permeability of membranes (Knobloch, Nelson, Köper, James, & McGillivray, 2015; Runas & Malmstadt, 2015). Whether the decrease in membrane potential is the cause or the result of the activation of mPTP opening is not clearly established. However, data from Rottenberg and Wu (1997) demonstrating that CsA restores mitochondrial potential in aging lymphocytes support the first hypothesis. Aging can also lower the threshold of potential necessary to mPTP opening and thus activate its opening and cell death.

Conversely, a mild decrease in mitochondrial potential caused by protonophores was shown to increase lifespan in yeast (Barros, Bandy, Tahara, & Kowaltowski, 2004), flies (Padalko, 2005) and mice (Caldeira da Silva, Cerqueira, Barbosa, Medeiros, & Kowaltowski, 2008). This is in accordance with data from Delaney et al. (2013) showing in yeast that cells with the lowest mitochondrial membrane potential have the longest subsequent replicative lifespan, but also with the demonstration that mild uncoupling protects mitochondrial function and contributes to the longevity of the most active human muscle fibers (Amara et al., 2007). A possible reason for this protective effect is the prevention of ROS production which is a well‐known consequence of a mild drop in membrane potential (Skulachev, 1998). Another explanation is the initiation of a mitochondrial retrograde response. The decline in potential activates beneficial changes in transcription resulting in increased lifespan (Miceli, Jiang, Tiwari, Rodriguez‐Quiñones, & Jazwinski, 2012). It must be mentioned that the reduction in membrane potential must be mild to avoid reaching the critical value for mPTP opening.

### 3.4 Nicotinamide adenine nucleotides, mPTP, and aging

Several data suggest that aging reduces cellular nicotinamide adenine dinucleotide (NAD^+^). This was observed in several organs in mice and in *Caenorhabditis elegans*. There is also evidence of NAD^+^ reduction in aged human tissues (Fang et al., 2014; Massudi et al., 2012; Mills et al., 2016; Mouchiroud et al., 2013; Zhang et al., 2016; Zhu, Lu, Lee, Ugurbil, & Chen, 2015). Conversely, supplementation with NAD^+^ precursors or overexpression of a NAD^+^ synthetic enzyme nicotinamidase have been reported to extend lifespan and to improve healthspan in different species (for review, see Fang et al., 2017). Conversion of NAD^+^ to NADH plays a key role in mitochondrial metabolism. NAD^+^ is reduced in NADH by four steps of the tricarboxylic acid cycle and during the oxidation of fatty and amino acids. NADH provides electrons to complex I of the respiratory chain to establish a protonmotive force which is responsible for ATP synthesis. A drop in NAD^+^ cellular levels can therefore limit NADH generation and consequently decrease mitochondrial membrane potential and oxidative phosphorylation. As previously mentioned, a drop in mitochondrial potential favors the frequency and the duration of mPTP opening which in turn can induce the release of NAD^+^ from mitochondria and thus contribute directly to its mitochondrial depletion during aging. In addition, the electron transport chain via NADH produces NAD^+^ and the decrease in mitochondrial NADH will contribute to the decline in mitochondrial NAD^+^. This decrease in NADH can also participate to mPTP opening as evidence shows that mitochondria are more susceptible to mPTP when the antioxidant power is exhausted (Kowaltowski, de Souza‐Pinto, Castilho, & Vercesi, 2009).

Another important consequence of mitochondrial NAD^+^ depletion is the inhibition of mitochondrial sirtuin (SIRT) activity, especially SIRT3. Sirtuins are NAD^+^‐dependent deacetylases that have been linked to lifespan prolongation in humans (Bellizzi et al., 2005) and involved in the response to nutritional and environmental perturbations such as DNA damage and oxidative stress (Sack & Finkel, 2012; Satoh, Stein, & Imai, 2011). SIRT3 plays a critical role in the protection of mitochondria and has received much attention for its role in aging (Ansari et al., 2017; Sadoshima, 2011). More particularly, SIRT3 was shown to deacetylate CypD, a component of the mPTP, and to inhibit mPTP opening, thereby reducing oxidative stress and slowing down cardiac aging (Hafner et al., 2010). There is therefore a discrepancy with the concomitant observations showing that chronic inhibition of mPTP opening in CypD^−/−^ mice is not associated with a decrease but rather to an enhancement of cardiac hypertrophy during aging (Elrod et al., 2010). A possible link between both studies was provided by Nguyen et al. (2013) who demonstrated using CypD^−/−^ mice that CypD could modulate mitochondrial protein acetylation and thus mitochondrial metabolic changes in addition to its mPTP regulating properties.

**Figure 2:**
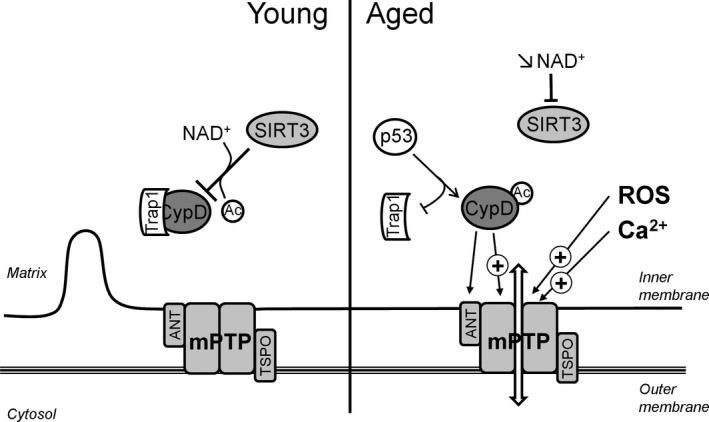
Age‐related alteration in cyclophilin D (CypD) regulation promotes mPTP opening. In mitochondria from young animals, CypD, the main regulator of mPTP, is inhibited by the Hsp90‐related mitochondrial matrix protein Trap1 and sirtuin 3 (SIRT3). CypD is no longer inhibited by association with Trap1 and deacetylation by sirtuin 3. The drop in NAD ^+^ pool inhibits SIRT3 deacetylase activity, and translocation of p53 activates CypD by displacing it from Trap1, favoring the translocation of the protein to the mPTP complex. ANT, adenine nucleotide translocase; TSPO, translocator protein; Ac, acetyl; ┴, inhibition; (+)→, stimulation

In summary, the decrease in mitochondrial NAD^+^ levels during aging inactivates SIRT3 and further stimulates mPTP opening, thus reinforcing mitochondrial dysfunction (Figure 2).

## 4 PUTATIVE MOLECULAR COMPONENTS OF mPTP AND AGING

Although mPTP activation is critical during the progression of aging, an important question is whether the putative structural components of the mPTP are concomitantly altered with age. The mPTP is a multiprotein complex whose molecular composition has evolved over time. As previously stated, genetic experiments have excluded some proteins such as VDAC, ANT, or TSPO from the main core of the pore and these proteins are now considered to rather have a regulatory role. Among the numerous components that have been proposed, only CypD is recognized as a real regulator of mPTP but it is not a structural pore component. Recent data propose ATP synthase as being the major component of the pore (Bernardi et al., 2015). This hypothesis is a priori counterintuitive as it seems opposite to the primary function of the enzyme, which is to produce energy, and furthermore requires a strict impermeability of the mitochondrial inner membrane. However, solid arguments support this hypothesis. The synthase could form a pore either by dimerization or by the detachment of the c ring subunit in association with the ANT, the phosphate carrier, and the CypD which binds to the oligomycin sensitivity conferral protein subunit of the enzyme (Bonora et al., 2017; Gerle, 2016; Giorgio et al., 2013).

### 4.1 ATP synthase and aging

Aging was shown to alter some properties of ATP synthase (Frenzel, Rommelspacher, Sugawa, & Dencher, 2010). Aging could decrease the maximal ATP synthase activity and impact the available ATP concentration in vivo but can also contribute to mPTP activation. An increased number of oxidized cysteine residues and a nitration of specific tyrosines was found in the ATP synthase of aged mouse hearts (Fernandez‐Sanz et al., 2015) and in the liver of aging rats (Haynes, Traaseth, Elfering, Fujisawa, & Giulivi, 2010), respectively.

Aging is also associated with post‐translationally modified isoforms of the enzyme which were found in three different model species of aging. Interestingly, a post‐translational modification of the oligomycin sensitivity conferral protein, which is considered as the ATP synthase binding target of CypD, has been identified in *Podospora anserina* (Groebe et al., 2007). This is in accordance with the progressive age‐dependent reorganization of the inner mitochondrial membrane observed in *Podospora anserine,* including disassembly of ATP synthase dimers and formation of contact sites between the inner and the outer membranes (Daum, Walter, Horst, Osiewacz, & Kühlbrandt, 2013; Figure 1). Importantly, the dissociation of ATP synthase dimers may involve CypD, suggesting a role for mPTP in this mitochondrial membrane reorganization.

### 4.2 CypD and aging

Besides ATP synthase, proteins regulating mPTP opening are modified by aging. CypD is enhanced in the brain mitochondria of old mice (Gauba, Guo, & Du, 2017) along with its interaction with its ATP synthase binding partner, the oligomycin sensitivity conferral protein, and this can mediate mPTP activation during aging. In contrast, partial deletion of CypD increases lifespan in mice (Vereczki et al., 2017)m emphasizing the role of the enzyme in aging. Post‐translational modifications of CypD have been suggested to play a role in aging. Oxidative stress observed in aged animals can alter the redox state of CypD, which is controlled by the thioredoxine system (Folda et al., 2016), and may lead to a more oxidized form of a critical site of CypD that may be responsible for mPTP activation (Nguyen et al., 2011). As discussed above, the inhibition of the deacetylation of CypD by SIRT3, resulting from the decrease in both NAD^+^ and SIRT3 levels in old animals, can also contribute to the enhancement of CypD activity and thus to mPTP opening (Hafner et al., 2010; Kwon, Kim, Lee, & Kim, 2015). In addition, SIRT3 regulates ROS‐mediated signaling as well as the detoxification of damaging ROS (Van de Ven, Santos, & Haigis, 2017). The decrease in SIRT3 can therefore indirectly amplify oxidative stress and CypD activity during aging (Figure 2).

CypD has also been shown to interact with p53 which is involved in the alteration of the cellular response occurring during aging. In response to oxidative stress, p53 accumulates in the mitochondrial matrix where it forms a complex with CypD and triggers mPTP opening (Vaseva et al., 2012). Recently, Lebedev et al. (2016) suggested a model in which p53 activates CypD by displacing it from Trap1, an Hsp90‐related mitochondrial matrix protein that complexes CypD, and maintains it inactive. This process may occur during aging and favors mPTP opening (Figure 2).

### 4.3 ANT and aging

CypD was also shown to interact with ANT which was reported to regulate mPTP opening (Crompton, Barksby, Johnson, & Capano, 2002; Woodfield, Rück, Brdiczka, & Halestrap, 1998). An increase in the ratio between CypD and ANT with aging was observed. This may contribute to higher susceptibility to mPTP opening (Marzetti et al., 2008) and to the reduction in affinity of ANT for CypD. An increased phospho‐GSK‐3β binding to ANT was suggested to be responsible for the inhibition of mPTP opening (Miura & Tanno, 2010; Nishihara et al., 2007). This mechanism would contribute to the cardioprotective effect of several drugs such as formononetin or resveratrol and of ischemic preconditioning (Cheng, Xia, Han, & Rong, 2016; Xi, Wang, Mueller, Norfleet, & Xu, 2009; Zhu, Rebecchi, Glass, Brink, & Liu, 2013; Zhu, Rebecchi, Wang, et al., 2013). In the aging heart, failure to reduce ANT/CypD interactions or decreased pGSK‐3β responsiveness of ANT could be responsible for the attenuation of cardioprotection afforded by ischemic preconditioning (Zhu, Rebecchi, Glass, et al., 2013; Zhu, Rebecchi, Wang, et al., 2013).

## 5 EVIDENCE FOR THE INVOLVEMENT OF mPTP OPENING IN AGE‐ASSOCIATED DISEASES

The incidence of pathology increases with age, and this is particularly marked for organs requiring a high and constant energy supply such as the heart, the brain, and the skeletal muscle but also the liver and the kidney. As mitochondria are the provider of energy of the cell, it is not surprising that mitochondrial dysfunction is considered as an important feature of aging (Tocchi, Quarles, Basisty, Gitari, & Rabinovitch, 2015). In this context, mPTP opening is believed to be involved in numerous age‐related disorders which are associated with a proapoptotic cellular environment (Ljubicic et al., 2010).

The incidence of myocardial infarction and heart failure increases with age. The aging heart is more susceptible to the damage induced by myocardial infarction, and most of the studies suggest that cardioprotection with ischemic or pharmacological conditioning becomes less effective (Boengler, Schulz, & Heusch, 2009; Fenton, Dickson, Meyer, & Dobson, 2000; Przyklenk, Maynard, Darling, & Whittaker, 2008; Schulman, Latchman, & Yellon, 2001). mPTP is thought to be a key factor in these processes, and the mechanism responsible for this loss of effectiveness may result from an activation of mPTP. Indeed, mPTP opening and cell death are increased in reperfused aged cardiomyocytes (Fernandez‐Sanz et al., 2015). This may be the consequence of the oxidative stress due to the increase in ROS production coupled with a decline in antioxidant defenses (Ferrara et al., 2008; Judge, Jang, Smith, & Hagen, 2005; Meng, Wong, Chen, & Ruan, 2007) prevailing in the aging heart. The observations that a mitochondria‐targeted ROS scavenger improved postischemic recovery of cardiac function (Escobales et al., 2014) and that the ROS scavenger Tempol restored pharmacological conditioning in aged rats (Zhu, Rebecchi, Glass, et al., 2013; Zhu, Rebecchi, Wang, et al., 2013) while preventing mPTP opening support this hypothesis.

mPTP opening also plays a role in the neuronal injury relevant to neurodegenerative diseases increasing in aging populations such as Alzheimer's, Parkinson's, and amyloid lateral sclerosis diseases **(**Angelova & Abramov, 2017; Du et al., 2008; Gandhi et al., 2009; Martin et al., 2009 **).** This is particularly true for Alzheimer's disease which is characterized by the presence of extracellular senile plaques, mainly composed of amyloid‐β (Aβ) peptide and intracellular neurofibrillary tangles made up of hyperphosphorylated tau protein (Selkoe, 2004). Several studies demonstrate that the Aβ peptide accumulates progressively into mitochondria (Hansson Petersen et al., 2008; Manczak et al., 2006) where it inhibits the activities of the respiratory chain complex and thus oxidative phosphorylation **(**Hernandez‐Zimbron et al., 2012; Lahmy, Long, Morin, Villard, & Maurice, 2015; Tillement, Lecanu, & Papadopoulos, 2011; Tsukada et al., 2014). The Aβ peptide can also potentially cause mPTP opening in vivo as it induces mitochondrial swelling, decreases mitochondrial membrane potential, and potentiates the effect of mPTP inducers in isolated brain mitochondria (Du et al., 2008; Moreira, Santos, Moreno, & Oliveira, 2001; Shevtzova, Kireeva, & Bachurin, 2001).This can be due to an indirect effect on the pore as the Aβ peptide has the ability to enhance intracellular calcium (Abramov, Canevari, & Duchen, 2004; Chin, Tse, Harris, & Jhamandas, 2006) and to induce oxidative stress (Lustbader et al., 2004; Reddy & Beal, 2008), which is increasingly recognized as a key factor in neurodegenerative disorders. These effects are possible mechanisms contributing to mPTP opening. Among the various enzymatic sources generating ROS, NADPH oxidase is an important contributor of Aβ peptide‐induced ROS (Abramov et al., 2004) and is considered as a common feature of neurodegenerative diseases. Whether this is a cause or a consequence of the neurodegenerative process remains questionable (Sorce et al., 2017).

Recent studies also implicate CypD in Aβ‐mediated mPTP. CypD binds to the Aβ peptide, and Aβ peptide–CypD complexes were isolated from patients with Alzheimer's disease and transgenic mice (Du, Guo, Zhang, Rydzewska, & Yan, 2011; Du et al., 2008). These complexes potentiate mitochondrial, neuronal, and synaptic stress, and genetic deletion of CypD protects the brain from Aβ‐induced neuronal degeneration (Du et al., 2008, 2014; Guo et al., 2013). Pharmacological inhibition of CypD by CsA was also shown to alleviate the deleterious effect of Aβ accumulation in isolated brain mitochondria (Moreira et al., 2001). Unfortunately, CsA and CsA derivatives lack clinical significance in Alzheimer's disease because of their side effects and of their poor blood–brain barrier permeability. However, neuronal CypD represents a promising therapeutic target for Alzheimer's disease and the development of nonpeptidic small molecule inhibitors of CypD is a promising approach (Ahmed‐Belkacem et al., 2016; Park et al., 2017).

Another promising drug target is the translocator protein (TSPO). Translocator protein is an outer mitochondrial membrane which has long been considered as a component of the mPTP and which regulates mitochondria‐mediated apoptotic cell death (Gatliff & Campanella, 2012; Morin, Musman, Pons, Berdeaux, & Ghaleh, 2016). Translocator protein expression is increased in elderly people and in patients with Alzheimer's disease (Kumar et al., 2012; Yasuno et al., 2008). Interestingly, inhibition of TSPO in drosophila inhibited apoptosis, extended fly lifespan, and inhibited Aβ peptide‐induced neurodegeneration (Lin et al., 2014). In addition, Elkamhawy et al. (2017) developed a novel class of TSPO ligands able to modulate Aβ peptide‐induced mPTP opening in hippocampal neuronal cell line.

mPTP opening might also be involved in other neurodegenerative diseases appearing with age. Indeed, CypD deletion studies show benefit in mouse models of amyloid lateral sclerosis and Parkinson's diseases as genetic ablation of CypD delayed the onset of disease and extended lifespan (Martin, Semenkow, Hanaford, & Wong, 2014; Martin et al., 2009), strengthening the role of the mPTP in the mechanisms of both diseases. The beneficial effect observed with a novel small mPTP inhibitor in a mouse model of amyloid lateral sclerosis confirmed that the mPTP could represent an interesting target for drug development in amyloid lateral sclerosis (Martin, Fancelli, et al., 2014). This is in line with the data of Keep, Elmér, Fong, and Csiszar (2001) who observed an improvement by CsA of the motion disorders in an amyloid lateral sclerosis mouse model.

A role of mPTP was also suggested in Huntington's disease (Short review: Quintanilla, Tapia, & Pérez, 2017). Indeed, expression of mutant huntingtin protein alters mitochondrial and cell viability through mPTP opening in striatal cells and cortical neurons (Quintanilla, Jin, von Bernhardi, & Johnson, 2013) and CsA showed protecting effects in a Huntington's disease mouse model (Kumar & Kumar, 2009). This idea was reinforced by the fact that CypD is upregulated in Huntington's patients and that this upregulation increased as Huntington's disease progressed (Shirendeb et al., 2011). However, other reports did not find such significant contribution of mPTP to mitochondrial injury in Huntington's disease, demonstrating that genetic inactivation of CypD does not modify the onset and the progression of the disease in mice (Brustovetsky et al., 2005; Pellman, Hamilton, Brustovetsky, & Brustovetsky, 2015; Perry et al., 2010).

Finally, it should be noted that the involvement of mPTP opening is not restricted to heart or brain age‐associated diseases. For instance, it was recently demonstrated that mitochondria are impaired in aging bone and that a CypD‐mPTP mechanism may be involved in aging‐related bone loss (Shum et al., 2016).

## 6 CONCLUSION

Life expectancy has greatly increased during the last 50 years, and logically, the number of elderly suffering from age‐related diseases has progressed concomitantly. It is therefore essential to understand the cellular mechanism of aging to improve the quality of life of the elderly and to apply strategies to fight against the pathologies appearing during aging. Several lines of evidence suggest that the mitochondrion, due to its multiple cellular functions, plays a critical role in aging and age‐related diseases. Mitochondrial bioenergetic dysfunction and its generation of damaging ROS were closely associated to aging and age‐related diseases. Recently, a large number of studies demonstrated that the mPTP, which is not definitely characterized at the molecular level, is more sensitive to opening in aged animals and in aging‐associated diseases and that its inhibition can enhance lifespan. This appears logical as the cellular modifications occurring during aging, that is, impaired calcium homeostasis, increased oxidative stress, oxidative modifications of proteins, enhancement of CypD level, and apoptosis, are factors contributing to and modulated by mPTP opening. However, doubts persist about the involvement of mPTP in the progression of aging and definitive experimental proofs of mPTP involvement have to be provided to demonstrate whether it is a cause or a consequence of aging. A better knowledge of the structural composition and of the regulation of the pore will probably help to elucidate the role of mPTP in longevity and healthspan.

## CONFLICT OF INTEREST

The authors report no conflict of interest.

## AUTHOR CONTRIBUTIONS

M.P. and D.M. wrote the manuscript. B.G. reviewed the manuscript.

## References

[acel12793-bib-0001] Abramov, A. Y. , Canevari, L. , & Duchen, M. R. (2004). Calcium signals induced by amyloid beta peptide and their consequences in neurons and astrocytes in culture. Biochimica et Biophysica Acta, 6(1742), 81–87. 10.1016/j.bbamcr.2004.09.006. 15590058

[acel12793-bib-0002] Ahmed‐Belkacem, A. , Colliandre, L. , Ahnou, N. , Nevers, Q. , Gelin, M. , Bessin, Y. , … Guichou, J. F. (2016). Fragment‐based discovery of a new family of non‐peptidic small‐molecule cyclophilin inhibitors with potent antiviral activities. Nature Communications, 7, 12777 10.1038/ncomms12777. PMC5036131 27652979

[acel12793-bib-0003] Allison, D. B. , Antoine, L. H. , Ballinger, S. W. , Bamman, M. M. , Biga, P. , Darley‐Usmar, V. M. , … Austad, S. N. (2014). Aging and energetics' ‘Top 40’ future research opportunities 2010–2013. F1000Research, 3, 219 10.12688/f1000research.5212.1 25324965 PMC4197746

[acel12793-bib-0004] Amara, C. E. , Shankland, E. G. , Jubrias, S. A. , Marcinek, D. J. , Kushmerick, M. J. , & Conley, K. E. (2007). Mild mitochondrial uncoupling impacts cellular aging in human muscles in vivo. Proceedings of the National Academy of Sciences of the United States of America, 104, 1057–1062. 10.1073/pnas.0610131104. 17215370 PMC1766336

[acel12793-bib-0005] Amigo, I. , Menezes‐Filho, S. L. , Luévano‐Martínez, L. A. , Chausse, B. , & Kowaltowski, A. J. (2017). Caloric restriction increases brain mitochondrial calcium retention capacity and protects against excitotoxicity. Aging Cell, 16, 73–81. 10.1111/acel.12527. 27619151 PMC5242290

[acel12793-bib-0006] Andreyev, A. Y. , Kushnareva, Y. E. , Murphy, A. N. , & Starkov, A. A. (2015). Mitochondrial ROS meta‐bolism: 10 years later. Biochemistry (Mosc), 80, 517–531. 10.1134/S0006297915050028. 26071769 PMC4511471

[acel12793-bib-0007] Andreyev, A. Y. , Kushnareva, Y. E. , & Starkov, A. A. (2005). Mitochondrial metabolism of reactive oxygen species. Biochemistry (Mosc), 70, 200–214. 10.1007/s10541-005-0102-7 15807660

[acel12793-bib-0008] Angelova, P. R. , & Abramov, A. Y. (2016). Functional role of mitochondrial reactive oxygen species in physiology. Free Radical Biology and Medicine, 100, 81–85. 10.1016/j.freeradbiomed.2016.06.005. 27296839

[acel12793-bib-0009] Angelova, P. R. , & Abramov, A. Y. (2017). Alpha‐synuclein and beta‐amyloid ‐ different targets, same players: Calcium, free radicals and mitochondria in the mechanism of neurodegeneration. Biochemical and Biophysical Research Communications, 483, 1110–1115. 10.1016/j.bbrc.2016.07.103. 27470584

[acel12793-bib-0010] Anisimov, V. N. , Bakeeva, L. E. , Egormin, P. A. , Filenko, O. F. , Isakova, E. F. , Manskikh, V. N. , … Skulachev, V. P. (2008). Mitochondria‐targeted plastoquinone derivatives as tools to interrupt execution of the aging program. 5. SkQ1 prolongs lifespan and prevents development of traits of senescence. Biochemistry (Mosc), 73, 1329–1342. 10.1134/S0006297908120055 19120018

[acel12793-bib-0011] Anisimov, V. N. , Egorov, M. V. , Krasilshchikova, M. S. , Lyamzaev, K. G. , Manskikh, V. N. , Moshkin, M. P. , … Skulachev, V. P. (2011). Effects of the mitochondria‐targeted antioxidant SkQ1 on lifespan of rodents. Aging (Albany, NY), 3, 1110–1119. 10.18632/aging.100404. 22166671 PMC3249456

[acel12793-bib-0012] Ansari, A. , Rahman, M. S. , Saha, S. K. , Saikot, F. K. , Deep, A. , & Kim, K. H. (2017). Function of the SIRT3 mitochondrial deacetylase in cellular physiology, cancer, and neurodegenerative disease. Aging Cell, 16, 4–16. 10.1111/acel.12538. 27686535 PMC5242307

[acel12793-bib-0013] Bae, Y. S. , Oh, H. , Rhee, S. G. , & Yoo, Y. D. (2011). Regulation of reactive oxygen species generation in cell signaling. Molecules and Cells, 32, 491–509. 10.1007/s10059-011-0276-3. 22207195 PMC3887685

[acel12793-bib-0014] Baines, C. P. , Kaiser, R. A. , Purcell, N. H. , Blair, N. S. , Osinska, H. , Hambleton, M. A. , … Molkentin, J. D. (2005). Loss of cyclophilin D reveals a critical role for mitochondrial permeability transition in cell death. Nature, 434, 658–662. 10.1038/nature03434. 15800627

[acel12793-bib-0015] Baines, C. P. , Kaiser, R. A. , Sheiko, T. , Craigen, W. J. , & Molkentin, J. D. (2007). Voltage‐dependent anion channels are dispensable for mitochondrial‐dependent cell death. Nature Cell Biology, 9, 550–555. 10.1038/ncb1575. 17417626 PMC2680246

[acel12793-bib-0016] Balaban, R. S. , Nemoto, S. , & Finkel, T. (2005). Mitochondria, oxidants, and aging. Cell, 120, 483–495. 10.1016/j.cell.2005.02.001. 15734681

[acel12793-bib-0017] Balasubramanian, P. , Howell, P. R. , & Anderson, R. M. (2017). Aging and caloric restriction research: A biological perspective with translational potential. EBioMedicine, 21, 37–44. 10.1016/j.ebiom.2017.06.015. 28648985 PMC5514430

[acel12793-bib-0018] Barros, M. H. , Bandy, B. , Tahara, E. B. , & Kowaltowski, A. J. (2004). Higher respiratory activity decreases mitochondrial reactive oxygen release and increases life span in *Saccharomyces cerevisiae* . Journal of Biological Chemistry, 279, 49883–49888. 10.1074/jbc.M408918200. 15383542

[acel12793-bib-0019] Bellizzi, D. , Rose, G. , Cavalcante, P. , Covello, G. , Dato, S. , De Rango, F. , … De Benedictis, G. (2005). A novel VNTR enhancer within the SIRT3 gene, a human homologue of SIR2, is associated with survival at oldest ages. Genomics, 85, 258–263. 10.1016/j.ygeno.2004.11.003. 15676284

[acel12793-bib-0020] Bernardi, P. , Rasola, A. , Forte, M. , & Lippe, G. (2015). The mitochondrial permeability transition pore: Channel formation by F‐ATP synthase, integration in signal transduction, and role in pathophysiology. Physiological Reviews, 95, 1111–1155. 10.1152/physrev.00001.2015. 26269524 PMC4600949

[acel12793-bib-0021] Besse, S. , Delcayre, C. , Chevalier, B. , Hardouin, S. , Heymes, C. , Bourgeois, F. , … Swynghedauw, B. (1994). Is the senescent heart overloaded and already failing? Cardiovascular Drugs and Therapy, 8, 581–587. 10.1007/BF00877412 7848894

[acel12793-bib-0022] Boengler, K. , Schulz, R. , & Heusch, G. (2009). Loss of cardioprotection with ageing. Cardiovascular Research, 83, 247–261. 10.1093/cvr/cvp033. 19176601

[acel12793-bib-0023] Bonora, M. , Morganti, C. , Morciano, G. , Pedriali, G. , Lebiedzinska‐Arciszewska, M. , Aquila, G. , … Pinton, P. (2017). Mitochondrial permeability transition involves dissociation of F1FO ATP synthase dimers and C‐ring conformation. EMBO Report, 18, 1077–1089. 10.15252/embr.201643602. PMC5494524 28566520

[acel12793-bib-0024] Brand, M. D. (2010). The sites and topology of mitochondrial superoxide production. Experimental Gerontology, 45, 466–472. 10.1016/j.exger.2010.01.003. 20064600 PMC2879443

[acel12793-bib-0025] Brand, M. D. (2014). The role of mitochondria in longevity and healthspan. Longevity & Healthspan, 3, 7 10.1186/2046-2395-3-7. 24855560 PMC4030464

[acel12793-bib-0026] Bratic, A. , & Larsson, N. G. (2013). The role of mitochondria in aging. Journal of Clinical Investigation, 123, 951–957. 10.1172/JCI64125. 23454757 PMC3582127

[acel12793-bib-0027] Brown, M. R. , Geddes, J. W. , & Sullivan, P. G. (2004). Brain region‐specific, age‐related, alterations in mitochondrial responses to elevated calcium. Journal of Bioenergetics and Biomembranes, 36, 401–406. 10.1023/B:JOBB.0000041775.10388.23. 15377879

[acel12793-bib-0028] Brustovetsky, N. , LaFrance, R. , Purl, K. J. , Brustovetsky, T. , Keene, C. D. , Low, W. C. , & Dubinsky, J. M. (2005). Age‐dependent changes in the calcium sensitivity of striatal mitochondria in mouse models of Huntington's disease. Journal of Neurochemistry, 93, 1361–1370. 10.1111/j.1471-4159.2005.03036.x. 15935052

[acel12793-bib-0029] Caldeira da Silva, C. C. , Cerqueira, F. M. , Barbosa, L. F. , Medeiros, M. H. , & Kowaltowski, A. J. (2008). Mild mitochondrial uncoupling in mice affects energy metabolism, redox balance and longevity. Aging Cell, 7, 552–560. 10.1111/j.1474-9726.2008.00407.x. 18505478

[acel12793-bib-0030] Chabi, B. , Ljubicic, V. , Menzies, K. J. , Huang, J. H. , Saleem, A. , & Hood, D. A. (2008). Mitochondrial function and apoptotic susceptibility in aging skeletal muscle. Aging Cell, 7, 2–12. 10.1111/j.1474-9726.2007.00347.x. 18028258

[acel12793-bib-0031] Cheng, Y. , Xia, Z. , Han, Y. , & Rong, J. (2016). Plant natural product formononetin protects rat cardiomyocyte H9c2 cells against oxygen glucose deprivation and reoxygenation via inhibiting ROS formation and promoting GSK‐3β phosphorylation. Oxidative Medicine and Cellular Longevity, 2016, 2060874 10.1155/2016/2060874. 27034732 PMC4806648

[acel12793-bib-0032] Chernyak, B. V. , & Bernardi, P. (1996). The mitochondrial permeability transition pore is modulated by oxidative agents through both pyridine nucleotides and glutathione at two separate sites. European Journal of Biochemistry, 238, 623–630. 10.1111/j.1432-1033.1996.0623w.x 8706660

[acel12793-bib-0033] Chin, J. H. , Tse, F. W. , Harris, K. , & Jhamandas, J. H. (2006). Beta‐amyloid enhances intracellular calcium rises mediated by repeated activation of intracellular calcium stores and nicotinic receptors in acutely dissociated rat basal forebrain neurons. Brain Cell Biology, 35, 173–186. 10.1007/s11068-007-9010-7. 17957482

[acel12793-bib-0034] Chouchani, E. T. , Methner, C. , Nadtochiy, S. M. , Logan, A. , Pell, V. R. , Ding, S. , … Murphy, M. P. (2013). Cardioprotection by S‐nitrosation of a cysteine switch on mitochondrial complex I. Nature Medicine, 19, 753–759. 10.1038/nm.3212. PMC4019998 23708290

[acel12793-bib-0035] Costantini, P. , Belzacq, A. S. , Vieira, H. L. , Larochette, N. , de Pablo, M. A. , Zamzami, N. , … Kroemer, G. (2000). Oxidation of a critical thiol residue of the adenine nucleotide translocator enforces Bcl‐2‐independent permeability transition pore opening and apoptosis. Oncogene, 19, 307–314. 10.1038/sj.onc.1203299. 10645010

[acel12793-bib-0036] Crompton, M. (1999). The mitochondrial permeability transition pore and its role in cell death. Biochemical Journal, 341, 233–249. 10.1042/bj3410233 10393078 PMC1220352

[acel12793-bib-0037] Crompton, M. , Barksby, E. , Johnson, N. , & Capano, M. (2002). Mitochondrial intermembrane junctional complexes and their involvement in cell death. Biochimie, 84, 143–152. 10.1016/S0300-9084(02)01368-8 12022945

[acel12793-bib-0038] Daum, B. , Walter, A. , Horst, A. , Osiewacz, H. D. , & Kühlbrandt, W. (2013). Age‐dependent dissociation of ATP synthase dimers and loss of inner‐membrane cristae in mitochondria. Proceedings of the National Academy of Sciences of the United States of America, 110, 15301–15306. 10.1073/pnas.1305462110. 24006361 PMC3780843

[acel12793-bib-0039] Del Campo, A. , Jaimovich, E. , & Tevy, M. F. (2016). Mitochondria in the aging muscles of flies and mice: New perspectives for old characters. Oxidative Medicine and Cellular Longevity, 2016, 9057593 10.1155/2016/9057593. 27630760 PMC5007348

[acel12793-bib-0040] Delaney, J. R. , Murakami, C. , Chou, A. , Carr, D. , Schleit, J. , Sutphin, G. L. , … Kaeberlein, M. (2013). Dietary restriction and mitochondrial function link replicative and chronological aging in *Saccharomyces cerevisiae* . Experimental Gerontology, 48, 1006–1013. 10.1016/j.exger.2012.12.001. 23235143 PMC3604125

[acel12793-bib-0041] Di Lisa, F. , Kaludercic, N. , Carpi, A. , Menabo, R. , & Giorgio, M. (2009). Mitochondrial pathways for ROS formation and myocardial injury: The relevance of p66(Shc) and monoamine oxidase. Basic Research in Cardiology, 104, 131–139. 10.1007/s00395-009-0008-4. 19242637

[acel12793-bib-0042] Dröge, W. (2002). Free radicals in the physiological control of cell function. Physiological Reviews, 82, 47–95. 10.1152/physrev.00018.2001. 11773609

[acel12793-bib-0043] Du, H. , Guo, L. , Fang, F. , Chen, D. , Sosunov, A. A. , McKhann, G. M. , … Yan, S. D. (2008). Cyclophilin D deficiency attenuates mitochondrial and neuronal perturbation and ameliorates learning and memory in Alzheimer's disease. Nature Medicine, 14, 1097–1105. 10.1038/nm.1868. PMC2789841 18806802

[acel12793-bib-0044] Du, H. , Guo, L. , Wu, X. , Sosunov, A. A. , McKhann, G. M. , Chen, J. X. , & Yan, S. S. (2014). Cyclophilin D deficiency rescues Aβ‐impaired PKA/CREB signaling and alleviates synaptic degeneration. Biochimica et Biophysica Acta, 1842, 2517–2527. 10.1016/j.bbadis.2013.03.004. 23507145 PMC3868643

[acel12793-bib-0045] Du, H. , Guo, L. , Zhang, W. , Rydzewska, M. , & Yan, S. (2011). Cyclophilin D deficiency improves mitochondrial function and learning/memory in aging Alzheimer disease mouse model. Neurobiology of Aging, 32, 398–406. 10.1016/j.neurobiolaging.2009.03.003. 19362755 PMC3304024

[acel12793-bib-0046] Duicu, O. M. , Mirica, S. N. , Gheorgheosu, D. E. , Privistirescu, A. I. , Fira‐Mladinescu, O. , & Muntean, D. M. (2013). Ageing‐induced decrease in cardiac mitochondrial function in healthy rats. Canadian Journal of Physiology and Pharmacology, 91, 593–600. 10.1139/cjpp-2012-0422. 23889593

[acel12793-bib-0047] Elkamhawy, A. , Park, J. E. , Hassan, A. H. E. , Pae, A. N. , Lee, J. , Park, B. G. , & Roh, E. J. (2017). Design, synthesis, biological evaluation and molecular modelling of 2‐(2‐aryloxyphenyl)‐1,4‐dihydroisoquinolin‐3(2H)‐ones: A novel class of TSPO ligands modulating amyloid‐β‐induced mPTP opening. European Journal of Pharmaceutical Sciences, 104, 366–381. https://doi.org/0.1016/j.ejps.2017.04.015. 28435076 10.1016/j.ejps.2017.04.015

[acel12793-bib-0048] Elrod, J. W. , Wong, R. , Mishra, S. , Vagnozzi, R. J. , Sakthievel, B. , Goonasekera, S. A. , & Molkentin, J. D. (2010). Cyclophilin D controls mitochondrial pore‐dependent Ca(2+) exchange, metabolic flexibility, and propensity for heart failure in mice. The Journal of Clinical Investigation, 120, 3680–3687. 10.1172/JCI43171. 20890047 PMC2947235

[acel12793-bib-0049] Escames, G. , López, A. , García, J. A. , García, L. , Acuña‐Castroviejo, D. , García, J. J. , & López, L. C. (2010). The role of mitochondria in brain aging and the effects of melatonin. Current Neuropharmacology, 8, 182–193. 10.2174/157015910792246245. 21358969 PMC3001212

[acel12793-bib-0050] Escobales, N. , Nuñez, R. E. , Jang, S. , Parodi‐Rullan, R. , Ayala‐Peña, S. , Sacher, J. R. , … Javadov, S. (2014). Mitochondria‐targeted ROS scavenger improves post‐ischemic recovery of cardiac function and attenuates mitochondrial abnormalities in aged rats. Journal of Molecular Cellular Cardiology, 77, 136–146. 10.1016/j.yjmcc.2014.10.009. 25451170 PMC4312194

[acel12793-bib-0051] Fang, E. F. , Lautrup, S. , Hou, Y. , Demarest, T. G. , Croteau, D. L. , Mattson, M. P. , & Bohr, V. A. (2017). NAD^+^ in aging: Molecular mechanisms and translational implications. Trends in Molecular Medicine, 23, 899–916. 10.1016/j.molmed.2017.08.001. 28899755 PMC7494058

[acel12793-bib-0052] Fang, E. F. , Scheibye‐Knudsen, M. , Brace, L. E. , Kassahun, H. , SenGupta, T. , Nilsen, H. , … Bohr, V. A. (2014). Defective mitophagy in XPA via PARP‐1 hyperactivation and NAD(+)/SIRT1 reduction. Cell, 157, 882–896. 10.1016/j.cell.2014.03.026. 24813611 PMC4625837

[acel12793-bib-0053] Fariss, M. W. , Chan, C. B. , Patel, M. , Van Houten, B. , & Orrenius, S. (2005). Role of mitochondria in toxic oxidative stress. Molecular Interventions, 5, 94–111. 10.1124/mi.5.2.7. 15821158

[acel12793-bib-0054] Fenton, R. A. , Dickson, E. W. , Meyer, T. E. , & Dobson, J. G. Jr (2000). Aging reduces the cardioprotective effect of ischemic preconditioning in the rat heart. Journal of Molecular Cellular Cardiology, 32, 1371–1375. 10.1006/jmcc.2000.1189. 10860777

[acel12793-bib-0055] Feridooni, H. , Dibb, K. M. , & Howlett, S. E. (2015). How cardiomyocyte excitation, calcium release and contraction become altered with age. Journal of Molecular Cellular Cardiology, 83, 62–72. 10.1016/j.yjmcc.2014.12.004. 25498213

[acel12793-bib-0056] Fernandez‐Sanz, C. , Ruiz‐Meana, M. , Castellano, J. , Miro‐Casas, E. , Nuñez, E. , Inserte, J. , … Garcia‐Dorado, D. (2015). Altered FoF1 ATP synthase and susceptibility to mitochondrial permeability transition pore during ischaemia and reperfusion in aging cardiomyocytes. Thrombosis and Haemostasis, 113, 441–451. 10.1160/TH14-10-0901. 25631625

[acel12793-bib-0057] Fernandez‐Sanz, C. , Ruiz‐Meana, M. , Miro‐Casas, E. , Nuñez, E. , Castellano, J. , Loureiro, M. , … Garcia‐Dorado, D. (2014). Defective sarcoplasmic reticulum‐mitochondria calcium exchange in aged mouse myocardium. Cell Death and Disease, 5, e1573 10.1038/cddis.2014.526. 25522267 PMC4454162

[acel12793-bib-0058] Ferrara, N. , Rinaldi, B. , Corbi, G. , Conti, V. , Stiuso, P. , Boccuti, S. , … Filippelli, A. (2008). Exercise training promotes SIRT1 activity in aged rats. Rejuvenation Research, 11, 139–150. 10.1089/rej.2007.0576. 18069916

[acel12793-bib-0059] Folda, A. , Citta, A. , Scalcon, V. , Calì, T. , Zonta, F. , Scutari, G. , … Rigobello, M. P. (2016). Mitochondrial thioredoxin system as a modulator of cyclophilin D redox state. Scientific Report, 6, 23071 10.1038/srep23071. PMC4791683 26975474

[acel12793-bib-0060] Frenzel, M. , Rommelspacher, H. , Sugawa, M. D. , & Dencher, N. A. (2010). Ageing alters the supramolecular architecture of OxPhos complexes in rat brain cortex. Experimental Gerontology, 45, 563–572. 10.1016/j.exger.2010.02.003. 20159033

[acel12793-bib-0061] Friberg, H. , & Wieloch, T. (2002). Mitochondrial permeability transition in acute neurodegeneration. Biochimie, 84, 241–250. 10.1016/S0300-9084(02)01381-0 12022955

[acel12793-bib-0062] Frolkis, V. V. , Frolkis, R. A. , Mkhitarian, L. S. , Shevchuk, V. G. , Fraifeld, V. E. , Vakulenko, L. G. , & Syrový, I. (1988). Contractile function and Ca^2+^ transport system of myocardium in ageing. Gerontology, 34, 64–74. 10.1159/000212932 2968294

[acel12793-bib-0063] Galluzzi, L. , Kepp, O. , Trojel‐Hansen, C. , & Kroemer, G. (2012). Mitochondrial control of cellular life, stress, and death. Circulation Research, 111, 1198–1207. 10.1161/CIRCRESAHA.112.268946. 23065343

[acel12793-bib-0064] Gandhi, S. , Wood‐Kaczmar, A. , Yao, Z. , Plun‐Favreau, H. , Deas, E. , Klupsch, K. , … Abramov, A. Y. (2009). PINK1‐associated Parkinson's disease is caused by neuronal vulnerability to calcium‐induced cell death. Molecular Cell, 33, 627–638. 10.1016/j.molcel.2009.02.013. 19285945 PMC2724101

[acel12793-bib-0065] García, N. , Zazueta, C. , Martínez‐Abundis, E. , Pavón, N. , & Chávez, E. (2009). Cyclosporin A is unable to inhibit carboxyatractyloside‐induced permeability transition in aged mitochondria. Comparative Biochemistry and Physiology Part C Toxicology & Pharmacology, 149, 374–381. 10.1016/j.cbpc.2008.09.006. 18835371

[acel12793-bib-0066] Gatliff, J. , & Campanella, M. (2012). The 18 kDa translocator protein (TSPO): A new perspective in mitochondrial biology. Current Molecular Medicine, 12, 356–368. 22364127 10.2174/1566524011207040356

[acel12793-bib-0067] Gauba, E. , Guo, L. , & Du, H. (2017). Cyclophilin D promotes brain mitochondrial F1FO ATP synthase dysfunction in aging mice. Journal of Alzheimers Disease, 55, 1351–1362. 10.3233/JAD-160822. PMC5496683 27834780

[acel12793-bib-0068] Genova, M. L. , & Lenaz, G. (2015). The interplay between respiratory supercomplexes and ROS in aging. Antioxidants and Redox Signaling, 23, 208–238. 10.1089/ars.2014.6214. 25711676

[acel12793-bib-0069] Gerle, C. (2016). On the structural possibility of pore‐forming mitochondrial FoF1 ATP synthase. Biochimica et Biophysica Acta, 1857, 1191–1196. 10.1016/j.bbabio.2016.03.008. 26968896

[acel12793-bib-0070] Giorgio, V. , von Stockum, S. , Antoniel, M. , Fabbro, A. , Fogolari, F. , Forte, M. , … Bernardi, P. (2013). Dimers of mitochondrial ATP synthase form the permeability transition pore. Proceedings of the National Academy of Sciences of the United States of America, 110, 5887–5892. 10.1073/pnas.1217823110. 23530243 PMC3625323

[acel12793-bib-0071] Go, Y. M. , & Jones, D. P. (2017). Redox theory of aging: Implications for health and disease. Clinical Science, 131, 1669–1688. 10.1042/CS20160897. 28667066 PMC5773128

[acel12793-bib-0072] Gonzalez‐Freire, M. , de Cabo, R. , Bernier, M. , Sollott, S. J. , Fabbri, E. , Navas, P. , & Ferrucci, L. (2015). Reconsidering the role of mitochondria in aging. The Journals of Gerontology, Series A: Biological Sciences and Medical Sciences, 70, 1334–1342. 10.1093/gerona/glv070. PMC4612387 25995290

[acel12793-bib-0073] Goodell, S. , & Cortopassi, G. (1998). Analysis of oxygen consumption and mitochondrial permeability with age in mice. Mechanisms of Ageing and Development, 101, 245–256. 10.1016/S0047-6374(97)00182-6 9622228

[acel12793-bib-0074] Gouspillou, G. , Sgarioto, N. , Kapchinsky, S. , Purves‐Smith, F. , Norris, B. , Pion, C. H. , … Hepple, R. T. (2014). Increased sensitivity to mitochondrial permeability transition and myonuclear translocation of endonuclease G in atrophied muscle of physically active older humans. The FASEB Journal, 28, 1621–1633. 10.1096/fj.13-242750. 24371120

[acel12793-bib-0075] Groebe, K. , Krause, F. , Kunstmann, B. , Unterluggauer, H. , Reifschneider, N. H. , Scheckhuber, C. Q. , … Schrattenholz, A. (2007). Differential proteomic profiling of mitochondria from *Podospora anserina*, rat and human reveals distinct patterns of age‐related oxidative changes. Experimental Gerontology, 42, 887–898. 10.1016/j.exger.2007.07.001. 17689904

[acel12793-bib-0076] Guo, L. , Du, H. , Yan, S. , Wu, X. , McKhann, G. M. , Chen, J. X. , & Yan, S. S. (2013). Cyclophilin D deficiency rescues axonal mitochondrial transport in Alzheimer's neurons. PLoS One, 8, e54914 10.1371/journal.pone.0054914. 23382999 PMC3561411

[acel12793-bib-0077] Gutiérrez‐Aguilar, M. , & Baines, C. P. (2015). Structural mechanisms of cyclophilin D‐dependent control of the mitochondrial permeability transition pore. Biochimica et Biophysica Acta, 1850, 2041–2047. 10.1016/j.bbagen.2014.11.009. 25445707 PMC4430462

[acel12793-bib-0078] Hafner, A. V. , Dai, J. , Gomes, A. P. , Xiao, C. Y. , Palmeira, C. M. , Rosenzweig, A. , & Sinclair, D. A. (2010). Regulation of the mPTP by SIRT3‐mediated deacetylation of CypD at lysine 166 suppresses age‐related cardiac hypertrophy. Aging (Albany, NY), 2, 914–923. 10.18632/aging.100252. 21212461 PMC3034180

[acel12793-bib-0079] Halestrap, A. P. (2010). A pore way to die: The role of mitochondria in reperfusion injury and cardioprotection. Biochemical Society Transactions, 38, 841–860. 10.1042/BST0380841. 20658967

[acel12793-bib-0080] Halestrap, A. P. , & Richardson, A. P. (2015). The mitochondrial permeability transition: A current perspective on its identity and role in ischaemia/reperfusion injury. Journal of Molecular and Cellular Cardiology, 78, 129–141. 10.1016/j.yjmcc.2014.08.018. 25179911

[acel12793-bib-0081] Halestrap, A. P. , Woodfield, K. Y. , & Connern, C. P. (1997). Oxidative stress, thiol reagents, and membrane potential modulate the mitochondrial permeability transition by affecting nucleotide binding to the adenine nucleotide translocase. Journal of Biological Chemistry, 272, 3346–3354. 10.1074/jbc.272.6.3346 9013575

[acel12793-bib-0082] Hansson Petersen, C. A. , Alikhani, N. , Behbahani, H. , Wiehager, B. , Pavlov, P. F. , Alafuzoff, I. , … Ankarcrona, M. (2008). The amyloid beta‐peptide is imported into mitochondria via the TOM import machinery and localized to mitochondrial cristae. Proceedings of the National Academy of Sciences of the United States of America, 105, 13145–13150. 10.1073/pnas.0806192105. 18757748 PMC2527349

[acel12793-bib-0083] Harman, D. (1972). The biologic clock: The mitochondria? Journal of the American Geriatrics Society, 20, 145–147. 10.1111/j.1532-5415.1972.tb00787.x 5016631

[acel12793-bib-0084] Hausenloy, D. , Wynne, A. , Duchen, M. , & Yellon, D. (2004). Transient mitochondrial permeability transition pore opening mediates preconditioning‐induced protection. Circulation, 109, 1714–1717. 10.1161/01.CIR.0000126294.81407.7D. 15066952

[acel12793-bib-0085] Haworth, R. A. , & Hunter, D. R. (1979). The Ca^2+^‐induced membrane transition in mitochondria. II. Nature of the Ca^2+^ trigger site. Archives of Biochemistry and Biophysics, 195, 460–467. 10.1016/0003-9861(79)90372-2 38751

[acel12793-bib-0086] Haynes, V. , Traaseth, N. J. , Elfering, S. , Fujisawa, Y. , & Giulivi, C. (2010). Nitration of specific tyrosines in FoF1 ATP synthase and activity loss in aging. American Journal of Physiology‐Endocrinology and Metabolism, 298, E978–E987. 10.1152/ajpendo.00739.2009. 20159857 PMC2867368

[acel12793-bib-0087] Hepple, R. T. (2016). Impact of aging on mitochondrial function in cardiac and skeletal muscle. Free Radical Biology and Medicine., 98, 177–186. 10.1016/j.freeradbiomed.2016.03.017. 27033952

[acel12793-bib-0088] Hernandez‐Zimbron, L. F. , Luna‐Muñoz, J. , Mena, R. , Vazquez‐Ramirez, R. , Kubli‐Garfias, C. , Cribbs, D. H. , … Gevorkian, G. (2012). Amyloid‐β peptide binds to cytochrome C oxidase subunit 1. PLoS One, 7, e42344 10.1371/journal.pone.0042344. 22927926 PMC3424232

[acel12793-bib-0089] Herraiz‐Martínez, A. , Álvarez‐García, J. , Llach, A. , Molina, C. E. , Fernandes, J. , Ferrero‐Gregori, A. , … Hove‐Madsen, L. (2015). Ageing is associated with deterioration of calcium homeostasis in isolated human right atrial myocytes. Cardiovascular Research, 106, 76–86. 10.1093/cvr/cvv046. 25712961 PMC4362404

[acel12793-bib-0090] Hofer, T. , Servais, S. , Seo, A. Y. , Marzetti, E. , Hiona, A. , Upadhyay, S. J. , … Leeuwenburgh, C. (2009). Bioenergetics and permeability transition pore opening in heart subsarcolemmal and interfibrillar mitochondria: Effects of aging and lifelong calorie restriction. Mechanisms of Ageing and Developement, 130, 297–307. 10.1016/j.mad.2009.01.004. PMC2680750 19428447

[acel12793-bib-0091] Hoffmann, B. , Stöckl, A. , Schlame, M. , Beyer, K. , & Klingenberg, M. (1994). The reconstituted ADP/ATP carrier activity has an absolute requirement for cardiolipin as shown in cysteine mutants. Journal of Biological Chemistry, 269, 1940–1944. 8294444

[acel12793-bib-0092] Hoppel, C. L. , Lesnefsky, E. J. , Chen, Q. , & Tandler, B. (2017). Mitochondrial dysfunction in cardiovascular aging. Advances in Experimental Medicine and Biology, 982, 451–464. 10.1007/978-3-319-55330-6_24. 28551802

[acel12793-bib-0093] Hunter, D. R. , & Haworth, R. A. (1979a). The Ca^2+^‐induced membrane transition in mitochondria. I. The protective mechanisms. Archives of Biochemistry and Biophysics, 195, 453–459. 10.1016/0003-9861(79)90371-0 383019

[acel12793-bib-0094] Hunter, D. R. , & Haworth, R. A. (1979b). The Ca^2+^‐induced membrane transition in mitochondria. III. Transitional Ca^2+^ release. Archives of Biochemistry and Biophysics, 195, 468–477. 10.1016/0003-9861(79)90373-4 112926

[acel12793-bib-0095] Jaeschke, H. , McGill, M. R. , & Ramachandran, A. (2012). Oxidant stress, mitochondria, and cell death mechanisms in drug‐induced liver injury: Lessons learned from acetaminophen hepatotoxicity. Drug Metabolism Reviews, 44, 88–106. 10.3109/03602532.2011.602688. 22229890 PMC5319847

[acel12793-bib-0096] Jahangir, A. , Ozcan, C. , Holmuhamedov, E. L. , & Terzic, A. (2001). Increased calcium vulnerability of senescent cardiac mitochondria: Protective role for a mitochondrial potassium channel opener. Mechanisms of Ageing and Development, 122, 1073–1086. 10.1016/S0047-6374(01)00242-1 11389925

[acel12793-bib-0097] Jahangir, A. , Sagar, S. , & Terzic, A. (2007). Aging and cardioprotection. Journal of Applied Physiology, 1985(103), 2120–2128. 10.1152/japplphysiol.00647.2007. 17717116

[acel12793-bib-0098] Judge, S. , Jang, Y. M. , Smith, A. , & Hagen, T. (2005). Leeuwenburgh C age‐associated increases in oxidative stress and antioxidant enzyme activities in cardiac interfibrillar mitochondria: Implications for the mitochondrial theory of aging. The FASEB Journal, 19, 419–421. 10.1096/fj.04-2622fje. 15642720

[acel12793-bib-0099] Kaplan, P. , Jurkovicova, D. , Babusikova, E. , Hudecova, S. , Racay, P. , Sirova, M. , … Krizanova, O. (2007). Effect of aging on the expression of intracellular Ca(2+) transport proteins in a rat heart. Molecular and Cellular Biochemistry., 301, 219–326. 10.1007/s11010-007-9414-9419. 17549608

[acel12793-bib-0100] Keep, M. , Elmér, E. , Fong, K. S. , & Csiszar, K. (2001). Intrathecal cyclosporin prolongs survival of late‐stage ALS mice. Brain Research, 894, 327–331. 10.1016/S0006-8993(01)02012-1 11251210

[acel12793-bib-0101] Kim, J. S. , He, L. , Qian, T. , & Lemasters, J. J. (2003). Role of the mitochondrial permeability transition in apoptotic and necrotic death after ischemia/reperfusion injury to hepatocytes. Current Molecular Medicine, 3, 527–535. 10.2174/1566524033479564 14527084

[acel12793-bib-0102] Klingenberg, M. (2009). Cardiolipin and mitochondrial carriers. Biochimica et Biophysica Acta, 1788, 2048–2058. 10.1016/j.bbamem.2009.06.007. 19539604

[acel12793-bib-0103] Knobloch, J. J. , Nelson, A. R. , Köper, I. , James, M. , & McGillivray, D. J. (2015). Oxidative damage to biomimetic membrane systems. In situ Fe(II)/ascorbate initiated oxidation and incorporation of synthetic oxidized phospholipids. Langmuir, 31, 12679–12687. 10.1021/acs.langmuir.5b02458. 26517192

[acel12793-bib-0104] Kohlhaas, M. , & Maack, C. (2013). Calcium release microdomains and mitochondria. Cardiovascular Research, 98, 259–268. 10.1093/cvr/cvt032. 23417042

[acel12793-bib-0105] Kokoszka, J. E. , Waymire, K. G. , Levy, S. E. , Sligh, J. E. , Cai, J. , Jones, D. P. , … Wallace, D. C. (2004). The ADP/ATP translocator is not essential for the mitochondrial permeability transition pore. Nature, 427, 461–465. 10.1038/nature02229. 14749836 PMC3049806

[acel12793-bib-0106] Kowaltowski, A. J. , de Souza‐Pinto, N. C. , Castilho, R. F. , & Vercesi, A. E. (2009). Mitochondria and reactive oxygen species. Free Radical Biology and Medicine, 47, 333–343. 10.1016/j.freeradbiomed.2009.05.004. 19427899

[acel12793-bib-0107] Krestinina, O. , Azarashvili, T. , Baburina, Y. , Galvita, A. , Grachev, D. , Stricker, R. , & Reiser, G. (2015). In aging, the vulnerability of rat brain mitochondria is enhanced due to reduced level of 2′,3′‐cyclic nucleotide‐3′‐phosphodiesterase (CNP) and subsequently increased permeability transition in brain mitochondria in old animals. Neurochemistry International, 80, 41–50. 10.1016/j.neuint.2014.09.008. 25277077

[acel12793-bib-0108] Kristal, B. S. , & Yu, B. P. (1998). Dietary restriction augments protection against induction of the mitochondrial permeability transition. Free Radical Biology and Medicine, 24, 1269–1277. 10.1016/S0891-5849(97)00444-9 9626583

[acel12793-bib-0109] Kumar, P. , & Kumar, A. (2009). Neuroprotective effect of cyclosporine and FK506 against 3‐nitropropionic acid induced cognitive dysfunction and glutathione redox in rat: Possible role of nitric oxide. Neuroscience Research., 63, 302–314. 10.1016/j.neures.2009.01.005 19367792

[acel12793-bib-0110] Kumar, A. , Muzik, O. , Shandal, V. , Chugani, D. , Chakraborty, P. , & Chugani, H. T. (2012). Evaluation of age‐related changes in translocator protein (TSPO) in human brain using (11)C‐[R]‐PK11195 PET. Journal of Neuroinflammation, 9, 232 10.1186/1742-2094-9-232. 23035793 PMC3546876

[acel12793-bib-0111] Kwak, H. B. , Song, W. , & Lawler, J. M. (2006). Exercise training attenuates age‐induced elevation in Bax/Bcl‐2 ratio, apoptosis, and remodeling in the rat heart. The FASEB Journal, 20, 791–793. 10.1096/fj.05-5116fje 16459353

[acel12793-bib-0112] Kwon, Y. Y. , Choi, K. M. , Cho, C. , & Lee, C. K. (2015). Mitochondrial efficiency‐dependent viability of *Saccharomyces cerevisiae* mutants carrying individual electron transport chain component deletions. Molecules and Cells, 38, 1054–1063. 10.14348/molcells.2015.0153. 26608359 PMC4696996

[acel12793-bib-0113] Kwon, Y. , Kim, J. , Lee, C. Y. , & Kim, H. (2015). Expression of SIRT1 and SIRT3 varies according to age in mice. Anatomy & Cell Biology., 48, 54–61. 10.5115/acb.2015.48.1.54. 25806122 PMC4371181

[acel12793-bib-0114] LaFrance, R. , Brustovetsky, N. , Sherburne, C. , Delong, D. , & Dubinsky, J. M. (2005). Age‐related changes in regional brain mitochondria from fischer 344 rats. Aging Cell, 4, 139–145. 10.1111/j.1474-9726.2005.00156.x. 15924570

[acel12793-bib-0115] Lahmy, V. , Long, R. , Morin, D. , Villard, V. , & Maurice, T. (2015). Mitochondrial protection by the mixed muscarinic/σ1 ligand ANAVEX2‐73, a tetrahydrofuran derivative, in Aβ25‐35 peptide‐injected mice, a nontransgenic Alzheimer's disease model. Frontiers in Cellular Neuroscience, 8, 463 10.3389/fncel.2014.00463. 25653589 PMC4299448

[acel12793-bib-0116] Lambert, A. J. , Boysen, H. M. , Buckingham, J. A. , Yang, T. , Podlutsky, A. , Austad, S. N. , … Brand, M. D. (2007). Low rates of hydrogen peroxide production by isolated heart mitochondria associate with long maximum lifespan in vertebrate homeotherms. Aging Cell, 6, 607–618. 10.1111/j.1474-9726.2007.00312.x. 17596208

[acel12793-bib-0117] Lebedev, I. , Nemajerova, A. , Foda, Z. H. , Kornaj, M. , Tong, M. , Moll, U. M. , & Seeliger, M. A. (2016). A novel in vitro CypD‐mediated p53 aggregation assay suggests a model for mitochondrial permeability transition by chaperone systems. Journal of Molecular Biology, 428, 4154–4167. 10.1016/j.jmb.2016.08.001. 27515399 PMC5453312

[acel12793-bib-0118] Li, H. , Zhou, C. , Chen, D. , Fang, N. , Yao, Y. , & Li, L. (2013). Failure to protect against myocardial ischemia‐reperfusion injury with sevoflurane postconditioning in old rats in vivo. Acta Anaesthesiologica Scandinavica, 57, 1024–1031. 10.1111/aas.12156. 23848060

[acel12793-bib-0119] Lin, R. , Angelin, A. , Da Settimo, F. , Martini, C. , Taliani, S. , Zhu, S. , & Wallace, D. C. (2014). Genetic analysis of dTSPO, an outer mitochondrial membrane protein, reveals its functions in apoptosis, longevity, and Ab42‐induced neurodegeneration. Aging Cell, 13, 507–518. 10.1111/acel.12200. 24977274 PMC4076708

[acel12793-bib-0120] Liu, L. , Zhu, J. , Brink, P. R. , Glass, P. S. , & Rebecchi, M. J. (2011). Age‐associated differences in the inhibition of mitochondrial permeability transition pore opening by cyclosporine A. Acta Anaesthesiologica Scandinavica, 55, 622–630. 10.1111/j.1399-6576.2011.02421.x. 21827445

[acel12793-bib-0121] Ljubicic, V. , Menzies, K. J. , & Hood, D. A. (2010). Mitochondrial dysfunction is associated with a pro‐apoptotic cellular environment in senescent cardiac muscle. Mechanisms of Ageing and Development, 131, 79–88. 10.1016/j.mad.2009.12.004. 20036683

[acel12793-bib-0122] López‐Otín, C. , Blasco, M. A. , Partridge, L. , Serrano, M. , & Kroemer, G. (2013). The hallmarks of aging. Cell, 153, 1194–1217. 10.1016/j.cell.2013.05.039. 23746838 PMC3836174

[acel12793-bib-0123] Lustbader, J. W. , Cirilli, M. , Lin, C. , Xu, H. W. , Takuma, K. , Wang, N. , … Wu, H. (2004). ABAD directly links Abeta to mitochondrial toxicity in Alzheimer's disease. Science, 2004(304), 448–452. 10.1126/science.1091230. 15087549

[acel12793-bib-0124] Manczak, M. , Anekonda, T. S. , Henson, E. , Park, B. S. , Quinn, J. , & Reddy, P. H. (2006). Mitochondria are a direct site of A beta accumulation in Alzheimer's disease neurons: Implications for free radical generation and oxidative damage in disease progression. Human Molecular Genetics, 15, 1437–1449. 10.1093/hmg/ddl066. 16551656

[acel12793-bib-0125] Marques‐Aleixo, I. , Rocha‐Rodrigues, S. , Santos‐Alves, E. , Coxito, P. M. , Passos, E. , Oliveira, P. J. , … Ascensão, A. (2012). In vitro salicylate does not further impair aging‐induced brain mitochondrial dysfunction. Toxicology, 302, 51–59. 10.1016/j.tox.2012.07.018. 22967791

[acel12793-bib-0126] Martin, L. J. , Fancelli, D. , Wong, M. , Niedzwiecki, M. , Ballarini, M. , Plyte, S. , & Chang, Q. (2014). GNX‐4728, a novel small molecule drug inhibitor of mitochondrial permeability transition, is therapeutic in a mouse model of amyotrophic lateral sclerosis. Frontiers in Cellular Neuroscience, 8, 433 10.3389/fncel.2014.00433. 25565966 PMC4271619

[acel12793-bib-0127] Martin, L. J. , Gertz, B. , Pan, Y. , Price, A. C. , Molkentin, J. D. , & Chang, Q. (2009). The mitochondrial permeability transition pore in motor neurons: Involvement in the pathobiology of ALS mice. Experimental Neurology, 218, 333–346. 10.1016/j.expneurol.2009.02.015. 19272377 PMC2710399

[acel12793-bib-0128] Martin, L. J. , Semenkow, S. , Hanaford, A. , & Wong, M. (2014). Mitochondrial permeability transition pore regulates Parkinson's disease development in mutant α‐synuclein transgenic mice. Neurobiology of Aging, 35, 1132–1152. 10.1016/j.neurobiolaging.2013.11.008. 24325796 PMC3948207

[acel12793-bib-0129] Marzetti, E. , Wohlgemuth, S. E. , Lees, H. A. , Chung, H. Y. , Giovannini, S. , & Leeuwenburgh, C. (2008). Age‐related activation of mitochondrial caspase‐independent apoptotic signaling in rat gastrocnemius muscle. Mechanisms of Ageing and Development, 129, 542–549. 10.1016/j.mad.2008.05.005. 18579179 PMC2585824

[acel12793-bib-0130] Massudi, H. , Grant, R. , Braidy, N. , Guest, J. , Farnsworth, B. , & Guillemin, G. J. (2012). Age‐associated changes in oxidative stress and NAD^+^ metabolism in human tissue. PLoS One, 7, e42357 10.1371/journal.pone.0042357. 22848760 PMC3407129

[acel12793-bib-0131] Mather, M. , & Rottenberg, H. (2000). Ageing enhances the activation of the permeability transition pore in mitochondria. Biochemical and Biophysical Research Communications, 273, 603–608. 10.1006/bbrc.2000.2994. 10873652

[acel12793-bib-0132] Maurel, A. , Hernandez, C. , Kunduzova, O. , Bompart, G. , Cambon, C. , Parini, A. , & Frances, B. (2003). Age‐dependent increase in hydrogen peroxide production by cardiac monoamine oxidase A in rats. American Journal of Physiology‐Heart and Circulatory Physiology, 284, H1460–H1467. 10.1152/ajpheart.00700.2002. 12531732

[acel12793-bib-0133] Menezes‐Filho, S. L. , Amigo, I. , Prado, F. M. , Ferreira, N. C. , Koike, M. K. , Pinto, I. F. D. , … Kowaltowski, A. J. (2017). Caloric restriction protects livers from ischemia/reperfusion damage by preventing Ca^2+^‐induced mitochondrial permeability transition. Free Radical Biology and Medicine, 110, 219–227. 10.1016/j.freeradbiomed.2017.06.013. 28642067

[acel12793-bib-0134] Meng, Q. , Wong, Y. T. , Chen, J. , & Ruan, R. (2007). Age‐related changes in mitochondrial function and antioxidative enzyme activity in fischer 344 rats. Mechanisms of Ageing and Development, 128, 286–292. 10.1016/j.mad.2006.12.008. 17270247

[acel12793-bib-0135] Miceli, M. V. , Jiang, J. C. , Tiwari, A. , Rodriguez‐Quiñones, J. F. , & Jazwinski, S. M. (2012). Loss of mitochondrial membrane potential triggers the retrograde response extending yeast replicative lifespan. Frontiers in Genetics, 2, 102 10.3389/fgene.2011.00102. 22303396 PMC3266616

[acel12793-bib-0136] Migliaccio, E. , Giorgio, M. , Mele, S. , Pelicci, G. , Reboldi, P. , Pandolfi, P. P. , … Pelicci, P. G. (1999). The p66shc adaptor protein controls oxidative stress response and life span in mammals. Nature, 18(402), 309–313. 10.1038/46311. 10580504

[acel12793-bib-0137] Mills, K. F. , Yoshida, S. , Stein, L. R. , Grozio, A. , Kubota, S. , Sasaki, Y. , … Imai, S. I. (2016). Long‐term administration of nicotinamide mononucleotide mitigates age‐associated physiological decline in mice. Cell Metabolism, 24, 795–806. 10.1016/j.cmet.2016.09.013. 28068222 PMC5668137

[acel12793-bib-0138] Miura, S. , Saitoh, S. I. , Kokubun, T. , Owada, T. , Yamauchi, H. , Machii, H. , & Takeishi, Y. (2017). Mitochondrial‐targeted antioxidant maintains blood flow, mitochondrial function, and redox balance in old mice following prolonged limb ischemia. International Journal of Molecular Sciences, 18(9), E1897 10.3390/ijms18091897. 28869535 PMC5618546

[acel12793-bib-0139] Miura, T. , & Tanno, M. (2010). Mitochondria and GSK‐3beta in cardioprotection against ischemia/reperfusion injury. Cardiovascular Drugs and Therapy, 24, 255–263. 10.1007/s10557-010-6234-z. 20490903

[acel12793-bib-0140] Moreira, P. I. , Santos, M. S. , Moreno, A. , & Oliveira, C. (2001). Amyloid beta‐peptide promotes permeability transition pore in brain mitochondria. Bioscience Reports, 21, 789–800. 10.1023/A:1015536808304 12166828

[acel12793-bib-0141] Morin, D. , Hauet, T. , Spedding, M. , & Tillement, J. (2001). Mitochondria as target for antiischemic drugs. Advanced Drug Delivery Reviews, 49, 151–174. 10.1016/j.addr.2017.11.004. 11377809

[acel12793-bib-0142] Morin, D. , Musman, J. , Pons, S. , Berdeaux, A. , & Ghaleh, B. (2016). Mitochondrial translocator protein (TSPO): From physiology to cardioprotection. Biochemical Pharmacology, 105, 1–13. 10.1016/j.bcp.2015.12.003. 26688086

[acel12793-bib-0143] Mouchiroud, L. , Houtkooper, R. H. , Moullan, N. , Katsyuba, E. , Ryu, D. , Cantó, C. , … Auwerx, J. (2013). The NAD(+)/sirtuin pathway modulates longevity through activation of mitochondrial UPR and FOXO signaling. Cell, 154, 430–441. 10.1016/j.cell.2013.06.016. 23870130 PMC3753670

[acel12793-bib-0144] Napoli, C. , Martin‐Padura, I. , de Nigris, F. , Giorgio, M. , Mansueto, G. , Somma, P. , … Pelicci, P. (2003). Deletion of the p66Shc longevity gene reduces systemic and tissue oxidative stress, vascular cell apoptosis, and early atherogenesis in mice fed a high‐fat diet. Proceedings of the National Academy of Sciences of the United States of America, 100, 2112–2116. 10.1073/pnas.0336359100. 12571362 PMC149967

[acel12793-bib-0145] Nguyen, T. T. , Stevens, M. V. , Kohr, M. , Steenbergen, C. , Sack, M. N. , & Murphy, E. (2011). Cysteine 203 of cyclophilin D is critical for cyclophilin D activation of the mitochondrial permeability transition pore. Journal of Biological Chemistry, 286, 40184–40192. 10.1074/jbc.M111.243469. 21930693 PMC3220546

[acel12793-bib-0146] Nguyen, T. T. , Wong, R. , Menazza, S. , Sun, J. , Chen, Y. , Wang, G. , … Murphy, E. (2013). Cyclophilin D modulates mitochondrial acetylome. Circulation Research, 113, 1308–1319. 10.1161/CIRCRESAHA.113.301867. 24062335 PMC4180423

[acel12793-bib-0147] Nickel, A. , Kohlhaas, M. , & Maack, C. (2014). Mitochondrial reactive oxygen species production and elimination. Journal of Molecular and Cellular Cardiology, 73, 26–33. 10.1016/j.yjmcc.2014.03.011. 24657720

[acel12793-bib-0148] Nishihara, M. , Miura, T. , Miki, T. , Tanno, M. , Yano, T. , Naitoh, K. , … Shimamoto, K. (2007). Modulation of the mitochondrial permeability transition pore complex in GSK‐3beta‐mediated myocardial protection. Journal of Molecular and Cellular Cardiology, 43, 564–570. 10.1016/j.yjmcc.2007.08.010. 17931653

[acel12793-bib-0149] Owada, T. , Yamauchi, H. , Saitoh, S. I. , Miura, S. , Machii, H. , & Takeishi, Y. (2017). Resolution of mitochondrial oxidant stress improves aged‐cardiovascular performance. Coronary Artery Disease, 28, 33–43. 10.1097/MCA.0000000000000434. 27740971 PMC5145249

[acel12793-bib-0150] Padalko, V. I. (2005). Uncoupler of oxidative phosphorylation prolongs the lifespan of Drosophila. Biochemistry (Mosc), 70, 986–989. 10.1007/s10541-005-0213-1 16266268

[acel12793-bib-0151] Pandya, J. D. , Grondin, R. , Yonutas, H. M. , Haghnazar, H. , Gash, D. M. , Zhang, Z. , & Sullivan, P. G. (2015). Decreased mitochondrial bioenergetics and calcium buffering capacity in the basal ganglia correlates with motor deficits in a nonhuman primate model of aging. Neurobiology of Aging, 36, 1903–1913. 10.1016/j.neurobiolaging.2015.01.018. 25726361

[acel12793-bib-0152] Paradies, G. , Paradies, V. , Ruggiero, F. M. , & Petrosillo, G. (2014). Cardiolipin and mitochondrial function in health and disease. Antioxidants & Redox Signaling, 20, 1925–1953. 10.1089/ars.2013.5280. 24094094

[acel12793-bib-0153] Paradies, G. , Paradies, V. , Ruggiero, F. M. , & Petrosillo, G. (2017). Mitochondrial bioenergetics decay in aging: Beneficial effect of melatonin. Cellular and Molecular Life Sciences, 74, 3897–3911. 10.1007/s00018-017-2619-5. 28785806 PMC11107727

[acel12793-bib-0154] Park, I. , Londhe, A. M. , Lim, J. W. , Park, B. G. , Jung, S. Y. , Lee, J. Y. , … Pae, A. N. (2017). Discovery of non‐peptidic small molecule inhibitors of cyclophilin D as neuroprotective agents in Aβ‐induced mitochondrial dysfunction. Journal of Computer‐Aided Molecular Design, 31, 929–941. 10.1007/s10822-017-0067-9. 28913661

[acel12793-bib-0155] Park, D. C. , & Yeo, S. G. (2013). Aging. Korean Journal of Audiology, 17, 39–44. 10.7874/kja.2013.17.2.39 24653904 PMC3936540

[acel12793-bib-0156] Payne, B. A. , & Chinnery, P. F. (2015). Mitochondrial dysfunction in aging: Much progress but many unresolved questions. Biochimica et Biophysica Acta, 1847, 1347–1353. 10.1016/j.bbabio.2015.05.022. 26050973 PMC4580208

[acel12793-bib-0157] Pellman, J. J. , Hamilton, J. , Brustovetsky, T. , & Brustovetsky, N. (2015). Ca(2+) handling in isolated brain mitochondria and cultured neurons derived from the YAC128 mouse model of Huntington's disease. Journal of Neurochemistry, 134, 652–667. 10.1111/jnc.13165. 25963273 PMC4516671

[acel12793-bib-0158] Perry, G. M. , Tallaksen‐Greene, S. , Kumar, A. , Heng, M. Y. , Kneynsberg, A. , van Groen, T. , … Lesort, M. (2010). Mitochondrial calcium uptake capacity as a therapeutic target in the R6/2 mouse model of Huntington's disease. Human Molecular Genetics, 19, 3354–3371. 10.1093/hmg/ddq247. 20558522 PMC2916705

[acel12793-bib-0159] Petronilli, V. , Miotto, G. , Canton, M. , Brini, M. , Colonna, R. , Bernardi, P. , & Di Lisa, F. (1999). Transient and long‐lasting openings of the mitochondrial permeability transition pore can be monitored directly in intact cells by changes in mitochondrial calcein fluorescence. Biophysical Journal, 76, 725–734. 10.1016/S0006-3495(99)77239-5. 9929477 PMC1300077

[acel12793-bib-0160] Petrosillo, G. , Casanova, G. , Matera, M. , Ruggiero, F. M. , & Paradies, G. (2006). Interaction of peroxidized cardiolipin with rat‐heart mitochondrial membranes: Induction of permeability transition and cytochrome c release. FEBS Letters, 580, 6311–6316. 10.1016/j.febslet.2006.10.036. 17083938

[acel12793-bib-0161] Petrosillo, G. , Moro, N. , Paradies, V. , Ruggiero, F. M. , & Paradies, G. (2010). Increased susceptibility to Ca(2+)‐induced permeability transition and to cytochrome C release in rat heart mitochondria with aging: Effect of melatonin. Journal of Pineal Research, 48, 340–346. 10.1111/j.1600-079X.2010.00758.x. 20345745

[acel12793-bib-0162] Picard, M. , Ritchie, D. , Thomas, M. M. , Wright, K. J. , & Hepple, R. T. (2011). Alterations in intrinsic mitochondrial function with aging are fiber type‐specific and do not explain differential atrophy between muscles. Aging Cell, 10, 1047–1055. 10.1111/j.1474-9726.2011.00745.x. 21933339

[acel12793-bib-0163] Picard, M. , Ritchie, D. , Wright, K. J. , Romestaing, C. , Thomas, M. M. , Rowan, S. L. , … Hepple, R. T. (2010). Mitochondrial functional impairment with aging is exaggerated in isolated mitochondria compared to permeabilized myofibers. Aging Cell, 9, 1032–1046. 10.1111/j.1474-9726.2010.00628.x. 20849523

[acel12793-bib-0164] Picard, M. , Wright, K. J. , Ritchie, D. , Thomas, M. M. , & Hepple, R. T. (2012). Mitochondrial function in permeabilized cardiomyocytes is largely preserved in the senescent rat myocardium. PLoS One, 7, e43003 10.1371/journal.pone.0043003. 22912774 PMC3415432

[acel12793-bib-0165] Pietrangelo, L. , D'Incecco, A. , Ainbinder, A. , Michelucci, A. , Kern, H. , Dirksen, R. T. , … Protasi, F. (2015). Age‐dependent uncoupling of mitochondria from Ca^2^⁺ release units in skeletal muscle. Oncotarget, 6, 35358–35371. 10.18632/oncotarget.6139. 26485763 PMC4742110

[acel12793-bib-0166] Przyklenk, K. , Maynard, M. , Darling, C. E. , & Whittaker, P. (2008). Aging mouse hearts are refractory to infarct size reduction with post‐conditioning. Journal of the American College of Cardiology, 51, 1393–1398. 10.1016/j.jacc.2007.11.070. 18387442

[acel12793-bib-0167] Quintanilla, R. A. , Jin, Y. N. , von Bernhardi, R. , & Johnson, G. V. (2013). Mitochondrial permeability transition pore induces mitochondria injury in Huntington disease. Molecular Neurodegeneration, 8, 45 10.1186/1750-1326-8-45. 24330821 PMC3878840

[acel12793-bib-0168] Quintanilla, R. A. , Tapia, C. , & Pérez, M. J. (2017). Possible role of mitochondrial permeability transition pore in the pathogenesis of Huntington disease. Biochemical and Biophysical Research Communications, 483, 1078–1083. 10.1016/j.bbrc.2016.09.054. 27638306

[acel12793-bib-0169] Rao, V. K. , Carlson, E. A. , & Yan, S. S. (2014). Mitochondrial permeability transition pore is a potential drug target for neurodegeneration. Biochimica et Biophysica Acta, 1842, 1267–1272. 10.1016/j.bbadis.2013.09.003. 24055979 PMC3991756

[acel12793-bib-0170] Rauen, U. , & de Groot, H. (2004). New insights into the cellular and molecular mechanisms of cold storage injury. Journal of Investigative Medicine, 52, 299–309. 10.1136/jim-52-05-29. 15551652

[acel12793-bib-0171] Reddy, P. H. , & Beal, M. F. (2008). Amyloid beta, mitochondrial dysfunction and synaptic damage: Implications for cognitive decline in aging and Alzheimer's disease. Trends in Molecular Medicine, 14, 45–53. 10.1016/j.molmed.2007.12.002. 18218341 PMC3107703

[acel12793-bib-0172] Rocha‐Rodrigues, S. , Santos‐Alves, E. , Coxito, P. M. , Marques‐Aleixo, I. , Passos, E. , Guimarães, J. T. , … Ascensão, A. (2013). Combined effects of aging and in vitro non‐steroid anti‐inflammatory drugs on kidney and liver mitochondrial physiology. Life Sciences, 93, 329–337. 10.1016/j.lfs.2013.07.004. 23872100

[acel12793-bib-0173] Rottenberg, H. , & Wu, S. (1997). Mitochondrial dysfunction in lymphocytes from old mice: Enhanced activation of the permeability transition. Biochemical and Biophysical Research Communications, 240, 68–74. 10.1006/bbrc.1997.7605. 9367884

[acel12793-bib-0174] Runas, K. A. , & Malmstadt, N. (2015). Low levels of lipid oxidation radically increase the passive permeability of lipid bilayers. Soft Matter, 11, 499–505. 10.1039/c4sm01478b. 25415555 PMC4477792

[acel12793-bib-0175] Sack, M. N. , & Finkel, T. (2012). Mitochondrial metabolism, sirtuins, and aging. Cold Spring Harbor Perspectives in Biology, 4(12), a013102 10.1101/cshperspect.a013102. 23209156 PMC3504438

[acel12793-bib-0176] Sadoshima, J. (2011). Sirt3 targets mPTP and prevents aging in the heart. Aging (Albany, NY), 3, 12–13. 10.18632/aging.100266. 21248376 PMC3047131

[acel12793-bib-0177] Sanz, A. (2016). Mitochondrial reactive oxygen species: Do they extend or shorten animal lifespan? Biochimica et Biophysica Acta, 1857, 1116–1126. 10.1016/j.bbabio.2016.03.018. 26997500

[acel12793-bib-0178] Sastre, J. , Pallardó, F. V. , Plá, R. , Pellín, A. , Juan, G. , O'Connor, J. E. , … Viña, J. (1996). Aging of the liver: Age‐associated mitochondrial damage in intact hepatocytes. Hepatology, 24, 1199–1205. 10.1002/hep.510240536. 8903398

[acel12793-bib-0179] Satoh, A. , Stein, L. , & Imai, S. (2011). The role of mammalian sirtuins in the regulation of metabolism, aging, and longevity. Handbook of Experimental Pharmacology, 206, 125–162. 10.1007/978-3-642-21631-2_7. 21879449 PMC3745303

[acel12793-bib-0180] Schlame, M. , & Greenberg, M. L. (2017). Biosynthesis, remodeling and turnover of mitochondrial cardiolipin. Biochimica et Biophysica Acta, 1862, 3–7. 10.1016/j.bbalip.2016.08.010. 27556952 PMC5125896

[acel12793-bib-0181] Schulman, D. , Latchman, D. S. , & Yellon, D. M. (2001). Effect of aging on the ability of preconditioning to protect rat hearts from ischemia‐reperfusion injury. American Journal of Physiology‐Heart and Circulatory Physiology, 281, H1630–H1636. 10.1152/ajpheart.2001.281.4.H1630 11557553

[acel12793-bib-0182] Scorrano, L. , Petronilli, V. , & Bernardi, P. (1997). On the voltage dependence of the mitochondrial permeability transition pore. A critical appraisal. Journal of Biological Chemistry, 9(272), 12295–12299. 10.1074/jbc.272.19.12295 9139672

[acel12793-bib-0183] Selkoe, D. J. (2004). Cell biology of protein misfolding: The examples of Alzheimer's and Parkinson's diseases. Nature Cell Biology, 6, 1054–1061. 10.1038/ncb1104-1054. 15516999

[acel12793-bib-0184] Sena, L. A. , & Chandel, N. S. (2012). Physiological roles of mitochondrial reactive oxygen species. Molecular Cell, 48, 158–167. 10.1016/j.molcel.2012.09.025. 23102266 PMC3484374

[acel12793-bib-0185] Shevtzova, E. F. , Kireeva, E. G. , & Bachurin, S. O. (2001). Effect of beta‐amyloid peptide fragment 25–35 on nonselective permeability of mitochondria. Bulletin of Experimental Biology and Medicine, 132, 1173–1176. 10.1023/A:1014559331402 12152879

[acel12793-bib-0186] Shirendeb, U. , Reddy, A. P. , Manczak, M. , Calkins, M. J. , Mao, P. , Tagle, D. A. , & Reddy, P. H. (2011). Abnormal mitochondrial dynamics, mitochondrial loss and mutant huntingtin oligomers in Huntington's disease: Implications for selective neuronal damage. Human Molecular Genetics, 20, 1438–1455. 10.1093/hmg/ddr024. 21257639 PMC3049363

[acel12793-bib-0187] Shum, L. C. , White, N. S. , Nadtochiy, S. M. , Bentley, K. L. , Brookes, P. S. , Jonason, J. H. , & Eliseev, R. A. (2016). Cyclophilin D knock‐out mice show enhanced resistance to osteoporosis and to metabolic changes observed in aging bone. PLoS One, 11, e0155709 10.1371/journal.pone.0155709. 27183225 PMC4868300

[acel12793-bib-0188] Skulachev, V. P. (1998). Uncoupling: New approaches to an old problem of bioenergetics. Biochimica et Biophysica Acta, 1363, 100–124. 10.1016/S0005-2728(97)00091-1 9507078

[acel12793-bib-0189] Skulachev, M. V. , & Skulachev, V. P. (2014). New data on programmed aging – slow phenoptosis. Biochemistry (Mosc), 79, 977–993. 10.1134/S0006297914100010. 25519058

[acel12793-bib-0190] Sorce, S. , Stocker, R. , Seredenina, T. , Holmdahl, R. , Aguzzi, A. , Chio, A. , … Jaquet, V. (2017). NADPH oxidases as drug targets and biomarkers in neurodegenerative diseases: What is the evidence? Free Radical Biology and Medicine, 112, 387–396. 10.1016/j.freeradbiomed.2017.08.006. 28811143

[acel12793-bib-0191] Sugrue, M. M. , & Tatton, W. G. (2001). Mitochondrial membrane potential in aging cells. Biological Signals and Receptors, 10, 176–188. 10.1159/000046886. 11351127

[acel12793-bib-0192] Szalai, G. , Csordás, G. , Hantash, B. M. , Thomas, A. P. , & Hajnóczky, G. (2000). Calcium signal transmission between ryanodine receptors and mitochondria. Journal of Biological Chemistry, 275, 15305–15313. 10.1074/jbc.275.20.15305 10809765

[acel12793-bib-0193] Tillement, L. , Lecanu, L. , & Papadopoulos, V. (2011). Alzheimer's disease: Effects of β‐amyloid on mitochondria. Mitochondrion, 11, 13–21. 10.1016/j.mito.2010.08.009. 20817045

[acel12793-bib-0194] Tocchi, A. , Quarles, E. K. , Basisty, N. , Gitari, L. , & Rabinovitch, P. S. (2015). Mitochondrial dysfunction in cardiac aging. Biochimica et Biophysica Acta, 1847, 1424–1433. 10.1016/j.bbabio.2015.07.009. 26191650 PMC4575872

[acel12793-bib-0195] Toescu, E. C. , & Vreugdenhil, M. (2010). Calcium and normal brain ageing. Cell Calcium, 47, 158–164. 10.1016/j.nlm.2010.11.008. 20045187

[acel12793-bib-0196] Toman, J. , & Fiskum, G. (2011). Influence of aging on membrane permeability transition in brain mitochondria. Journal of Bioenergetics and Biomembranes, 43, 3–10. 10.1007/s10863-011-9337-8. 21311961 PMC4085790

[acel12793-bib-0197] Tsukada, H. , Nishiyama, S. , Ohba, H. , Kanazawa, M. , Kakiuchi, T. , & Harada, N. (2014). Comparing amyloid‐β deposition, neuroinflammation, glucose metabolism, and mitochondrial complex I activity in brain: A PET study in aged monkeys. European Journal of Nuclear Medicine and Molecular Imaging, 41, 2127–2136. 10.1007/s00259-014-2821-8. 24919653

[acel12793-bib-0198] Van de Ven, R. A. H. , Santos, D. , & Haigis, M. C. (2017). Mitochondrial sirtuins and molecular mechanisms of aging. Trends in Molecular Medicine, 23, 320–331. 10.1016/j.molmed.2017.02.005. 28285806 PMC5713479

[acel12793-bib-0199] Vaseva, A. V. , Marchenko, N. D. , Ji, K. , Tsirka, S. E. , Holzmann, S. , & Moll, U. M. (2012). p53 opens the mitochondrial permeability transition pore to trigger necrosis. Cell, 149, 1536–1548. 10.1016/j.cell.2012.05.014. 22726440 PMC3383624

[acel12793-bib-0200] Vereczki, V. , Mansour, J. , Pour‐Ghaz, I. , Bodnar, I. , Pinter, O. , Zelena, D. , … Chinopoulos, C. (2017). Cyclophilin D regulates lifespan and protein expression of aging markers in the brain of mice. Mitochondrion, 34, 115–126. 10.1016/j.mito.2017.03.003. 28288917

[acel12793-bib-0201] Wang, S. B. , Murray, C. I. , Chung, H. S. , & Van Eyk, J. E. (2013). Redox regulation of mitochondrial ATP synthase. Trends in Cardiovascular Medicine, 23, 14–18. 10.1016/j.tcm.2012.08.005. 23312134 PMC3936247

[acel12793-bib-0202] Woodfield, K. , Rück, A. , Brdiczka, D. , & Halestrap, A. P. (1998). Direct demonstration of a specific interaction between cyclophilin‐D and the adenine nucleotide translocase confirms their role in the mitochondrial permeability transition. Biochemical Journal, 336, 287–290. 10.1042/bj3360287 9820802 PMC1219869

[acel12793-bib-0203] Xi, J. , Wang, H. , Mueller, R. A. , Norfleet, E. A. , & Xu, Z. (2009). Mechanism for resveratrol‐induced cardioprotection against reperfusion injury involves glycogen synthase kinase 3beta and mitochondrial permeability transition pore. European Journal of Pharmacology, 604, 111–116. 10.1016/j.ejphar.2008.12.024. 19135050 PMC2861585

[acel12793-bib-0204] Yan, L. J. , & Sohal, R. S. (1998). Mitochondrial adenine nucleotide translocase is modified oxidatively during aging. Proceedings of the National Academy of Sciences of the United States of America, 95, 12896–12901. 10.1073/pnas.95.22.12896 9789011 PMC23645

[acel12793-bib-0205] Yasuno, F. , Ota, M. , Kosaka, J. , Ito, H. , Higuchi, M. , Doronbekov, T. K. , … Suhara, T. (2008). Increased binding of peripheral benzodiazepine receptor in Alzheimer's disease measured by positron emission tomography with [11C]DAA1106. Biological Psychiatry, 64, 835–841. 10.1016/j.biopsych.2008.04.021. 18514164

[acel12793-bib-0206] Zhang, H. , Ryu, D. , Wu, Y. , Gariani, K. , Wang, X. , Luan, P. , … Auwerx, J. (2016). NAD⁺ repletion improves mitochondrial and stem cell function and enhances life span in mice. Science, 352, 1436–1443. 10.1126/science.aaf2693. 27127236

[acel12793-bib-0207] Zhu, X. H. , Lu, M. , Lee, B. Y. , Ugurbil, K. , & Chen, W. (2015). In vivo NAD assay reveals the intracellular NAD contents and redox state in healthy human brain and their age dependences. Proceedings of the National Academy of Sciences of the United States of America., 112, 2876–2881. 10.1073/pnas.1417921112. 25730862 PMC4352772

[acel12793-bib-0208] Zhu, J. , Rebecchi, M. J. , Glass, P. S. , Brink, P. R. , & Liu, L. (2011). Cardioprotection of the aged rat heart by GSK‐3beta inhibitor is attenuated: Age‐related changes in mitochondrial permeability transition pore modulation. American Journal of Physiology‐Heart and Circulatory Physiology, 300, H922–H930. 10.1152/ajpheart.00860.2010. 21217064

[acel12793-bib-0209] Zhu, J. , Rebecchi, M. J. , Glass, P. S. , Brink, P. R. , & Liu, L. (2013). Interactions of GSK‐3β with mitochondrial permeability transition pore modulators during preconditioning: Age‐associated differences. The Journals of Gerontology, Series A: Biological Sciences and Medical Sciences, 68, 395–403. 10.1093/gerona/gls205. 23070879

[acel12793-bib-0210] Zhu, J. , Rebecchi, M. J. , Tan, M. , Glass, P. S. , Brink, P. R. , & Liu, L. (2010). Age‐associated differences in activation of Akt/GSK‐3beta signaling pathways and inhibition of mitochondrial permeability transition pore opening in the rat heart. The Journals of Gerontology, Series A: Biological Sciences and Medical Sciences, 65, 611–619. 10.1093/gerona/glq035. 20427381

[acel12793-bib-0211] Zhu, J. , Rebecchi, M. J. , Wang, Q. , Glass, P. S. , Brink, P. R. , & Liu, L. (2013). Chronic tempol treatment restores pharmacological preconditioning in the senescent rat heart. American Journal of Physiology‐Heart and Circulatory Physiology, 304, H649–H659. 10.1152/ajpheart.00794.2012. 23275621

